# Padding interpolation, median imputation, RobustScalar, and particle swarm optimization with heterogeneous classifiers: a robust combination for effective heart disease diagnosis

**DOI:** 10.3389/fmed.2025.1721740

**Published:** 2026-01-12

**Authors:** Sanjay Dhanka, Ankur Kumar, Surita Maini, Nitin Kumar, Jeewan Singh, Mudassir Khan, Mohamed Abbas, Amel Ksibi

**Affiliations:** 1 Department of Electrical Engineering, Graphic Era (Deemed to be University), Clement Town, Dehradun, India; 2 School of Computing and Electrical Engineering (SCEE), Indian Institute of Technology (IIT), Mandi, Himachal Pradesh, India; 3 Department of Electrical and Instrumentation Engineering, Sant Longowal Institute of Engineering and Technology, Sangrur, Punjab, India; 4 Centre for Research Impact & Outcome, Chitkara University Institute of Engineering and Technology, Chitkara University, Rajpura, India; 5 Department of Mechanical Engineering, Noida Institute of Engineering and Technology, Greater Noida, India; 6 University Centre for Research and Development, Chandigarh University, Mohali, Punjab, India; 7 Department of Computer Science, College of Computer Science, Applied College Tanumah, King Khalid University, Abha, Saudi Arabia; 8 Jadara University Research Center, Jadara University, Irbid, Jordan; 9 Electrical Engineering Department, College of Engineering, King Khalid University, Abha, Saudi Arabia; 10 Department of Information Systems, College of Computer and Information Sciences, Princess Nourah bint Abdulrahman University, Riyadh, Saudi Arabia

**Keywords:** machine learning classification, heart disease, particle swarm optimization, interpolation, imputation

## Abstract

**Introduction:**

Heart disease is a leading cause of death worldwide, necessitating accurate early diagnosis. Although machine learning (ML) shows potential for this task, many current models are hindered by data inconsistencies, poor feature selection, and limited robustness.

**Methods:**

This study proposes a novel, robust diagnostic framework. It employs advanced data preprocessing using Padding Interpolation for missing values, Median Imputation for outliers, and RobustScalar for scaling to ensure data integrity. A key innovation is an Improved Particle Swarm Optimization (IPSO) algorithm, enhanced with dynamic inertia weight and a mutation operator to avoid premature convergence. This IPSO performs dual optimization: selecting optimal features and tuning the hyperparameters of five classifiers (Logistic Regression, Linear Discriminant Analysis, Gaussian Naïve Bayes, Support Vector Classifier, and XGBoost).

**Results:**

The framework was evaluated on a composite dataset from five public repositories. The proposed IPSO-optimized XGBoost model achieved superior performance at a 90:10 train-test split, with an accuracy of 91.3%, sensitivity of 88.37%, specificity of 93.88%, precision of 92.68%, F1-score of 90.48%, and a Diagnostic Odds Ratio of 116.53. Statistical tests (p < 0.05) confirmed these improvements over baselines were significant. The model also demonstrated consistent generalizability on independent Cleveland and Statlog datasets.

**Discussion:**

The results establish that the integrated framework of rigorous preprocessing and the hybrid IPSO optimization-classification model creates a highly effective and generalizable pipeline for automated heart disease diagnosis, addressing key limitations of existing approaches.

## Graphical Abstract

**Figure:**
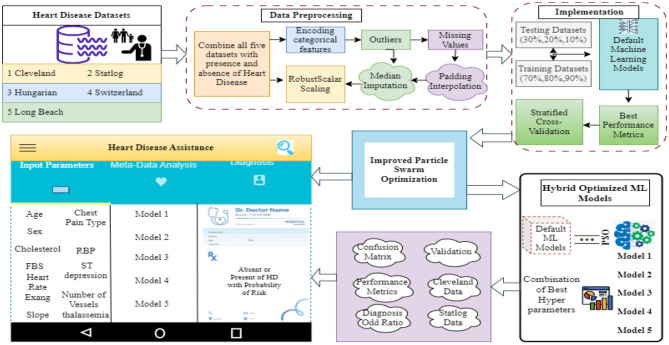
Hybrid Advanced Machine Learning Models.

## Highlights

- Thoroughly analyzed the five heterogeneous classifiers on three training sets.
- Introduced padding interpolation, median imputation, and RobustScalar methods.
- Particle swarm optimization is used for feature selection and weight optimization.
- Proposed hybrid optimized diagnostic models with promising and competitive results.
- Proposed models achieved consistent performance on unknown datasets.

## 1 Introduction

Heart disease (HD) encompasses a wide range of conditions affecting the heart and blood vessels, such as coronary artery disease, arrhythmia, heart failure, and valvular diseases. It is characterized by symptoms such as shortness of breath, fatigue, swelling of legs and feet, palpitations, chronic cough, weight gain, and irregular heartbeats (1). The risk of this disease growing is associated with certain leading factors, i.e., age, smoking, obesity, high cholesterol level, thalassemia, anxiety, excessive consumption of alcohol, high blood pressure, stress, and many others (2). Usually, patients with HD end up dying unless potent preventive measures are taken. The World Health Organization (WHO) has estimated that more than 18 million people die from HD each year, which is 32% of all global deaths (3). This makes HD the leading cause of death globally. One in four people under the age of 40 will have a heart attack. This risk increases drastically to double by the age of 50. Identifying people who are at risk of HD early and providing them with appropriate treatment can help to reduce the number of deaths (4, 5).

In the present scenario, cardiologists consider angiography (used to visualize any blockages or narrowing of the arteries), electrocardiogram (used to detect abnormalities in the heart's rhythm), and echocardiogram (used to assess the size and function of the heart or any structural abnormalities) to be the most appropriate methods for diagnosing HD (6). These methods are expensive, require specialized training in a particular instrument, and can have serious side effects. Moreover, it is challenging to manually examine the likelihood of developing HD at an early stage based on risk factors. The challenges associated with current methods of predicting HD highlight the need for the development of low-cost, non-invasive, and automated technologies for the prediction of HD in the initial stage. Fortunately, machine learning (ML) techniques offer a promising solution for meeting the above challenges of predicting HD (7). These ML techniques can be used to develop economical, non-invasive, and fully automated methods with high precision for predicting HD. ML is a subset of Artificial Intelligence (AI) that can be used to learn from patient data, such as medical history, risk factors, and laboratory tests, to detect whether a patient is likely to have HD. It involves several learning concepts, such as supervised learning (SL), unsupervised learning (UL), and semi-supervised Learning (SSL) (8).

In these ML techniques, particularly the SL ones, the primary task is to classify a given subject, whether it is suffering from HD or not. This is attained by training these techniques on a labeled HD dataset to be subsequently employed for classification. The UL techniques use cluster analysis with dimensional reduction for unlabeled datasets, and SSL combines SL and UL techniques. The increasing availability of public clinical data repositories has made it easier than ever to access electronic data of patients for further assessment. This publicly available data is being used to develop a variety of ML intelligent systems for medical applications, such as skin disease diagnosis (9), epilepsy EEG signals classification (10), tissue engineering (11), cancer field (12), early disease prediction field (13), drug delivery system (14), and heart disease prediction (15).

In the current scenario, clinical dataset management and prediction of HD at an early stage have become a crucial task in the medical field. The careful entry of patient data, incorporating a variety of attributes and diagnostics, and history related to the disease, is essential for delivering high-quality medical facilities. Otherwise, clinical data mining would be challenging due to the possibility of missing values and incorrect data format. It is also important to have fine dataset management before applying classification techniques since this might affect the outcomes. When datasets are precise, robust, trustworthy, and devoid of noise, HD classification becomes faster and easier. The refined dataset can be acquired with the help of preprocessing and feature engineering techniques (16). The former techniques include data refining processes, such as handling missing values, outliers, and noise, whereas feature engineering checks the correlation of all features with the target and transforms the original dataset into a particular scale (17).

Although algorithms for the diagnosis of HD have yielded promising results, there is still room for upgradation, and hence, research in this area is still ongoing. The following section provides a critical analysis of recent literature, highlighting their methodologies, strengths, and limitations.

### 1.1 Critical analysis of related work

#### 1.1.1 Studies focusing on ensemble and hybrid models

For instance, Ozcan et al. (6) proposed a classification and regression tree (CART) approach to predict HD based on the decision influenced by the rank of the features. The experimental results on the combined five different datasets, i.e., Cleveland, Statlog, Hungarian, Switzerland, and Long Beach, claimed better performance than the existing models. However, the model lacks advanced optimization for feature selection and may not capture complex, non-linear relationships as effectively as ensemble methods. Bhatt et al. (18) employed a method of k-modes clustering with the Huang starting technique to further improve the classification of HD. The simulation results on a publicly accessible HD dataset proved that their established method resulted in better performance than other classifiers, i.e., multi-layer perceptron, random forest, and decision tree. A key limitation is that it was not benchmarked on standard UCI datasets like Cleveland, limiting direct comparability. Hera et al. (19) developed a multi-tier ensemble (MTE) technique with random forest feature selection for efficient diagnosis of HD. In this research study, the MTE model consists of several combinations of ML and ensemble learning techniques. It is applied on the Cleveland and Statlog datasets to confirm the effectiveness of the method based on several performance metrics, that is, recall, accuracy, precision, and f-measure. While effective, the ensemble structure can be computationally expensive, and performance varies across datasets. Rani et al. (20) proposed a hybrid decision support system, that is, a combination of a genetic algorithm, a recursive feature elimination method, and ML models that can support the early prediction of HD based on the clinical constraints of the patient. They used the synthetic minority oversampling technique for data balancing problems and analyzed the better performance results with a random forest classifier on the Cleveland HD dataset. The study was focused on a single dataset and lacked validation on more challenging datasets, like Hungarian.

Zhenya et al. (1) developed a cost sensitivity ensemble method to reduce the misclassification cost in the prediction of HD. In this ensemble method, the authors used five heterogeneous classifiers, that is, random forest, extreme learning machine, logistic regression (LR), support vector machine (SVM), and k-nearest neighbor. They used a *t*-test to explore the performance of this ensemble method and obtained better results than individual classifiers and existing studies. A gap identified was the model's sensitivity to class distribution, with lower performance on the imbalanced Hungarian dataset. Mohan et al. (21) developed a hybrid random forest with a linear model (HRFLM) for effective HD prediction by using 13 significant clinical features. The proposed model was implemented on the Cleveland HD dataset by using R Studio Rattle platform and exhibited higher performance than the nine other models. Although high sensitivity was achieved, the model was not tested for generalization on other HD datasets, and high sensitivity might come at the cost of a higher false positive rate.

#### 1.1.2 Studies utilizing optimization algorithms

Koppu et al. (22) proposed a fitness-oriented dragonfly algorithm to minimize the classification errors and fed the resultant features into the deep belief framework. The proposed approach was employed on the Cleveland and Statlog datasets, and the performance was evaluated over other traditional models such as gray wolf optimization, particle swarm optimization, firefly, and dragonfly algorithm. A significant drawback was the very low precision (30.98%) on the Cleveland dataset, indicating that class imbalance was not adequately handled. Tama et al. (23) employed an HD detection system using a two-tier classifier method. This method was implemented on various heart disease datasets, such as Z-Alizadeh Sani, Statlog, Hungarian, and Cleveland. In this study, the authors used particle swarm optimization techniques to select the most significant feature set and adopted a twofold statistical test to validate the hypothesis. This study proves that the proposed model adds a significant contribution compared to the existing published study. However, the two-tier model adds complexity, making the decision-making process less interpretable for clinicians. Amin et al. (24) proposed an efficient HD prediction approach, that is, Vote, to identify the significant features with improved performance. This approach used an ensemble technique of Naïve Bayes and logistic regression. The simulation results were performed using various combinations of features and seven classification techniques.

Khourdifi et al. (25) employed an Artificial Neural Network, which is optimized by two metaheuristic techniques, that is, PSO and Ant Colony Optimization. In this study, used fast correlation-based feature selection technique to filter redundant features in order to enhance the quality of HD prediction. This hybrid approach was applied to the Cleveland HD dataset, and the classification results were compared with the aforementioned methods. The study demonstrated the potential of hybrid metaheuristics but did not focus on advanced data preprocessing for outlier handling. Vijayashree et al. (26) proposed an improved fitness function of PSO with the help of the support vector machine (SVM) algorithm. In this method, the PSO technique was used to select the most significant features presented in the dataset and find the best solution in less time. With the help of SVM, the velocity and position of the PSO particles were updated and the results were compared with various existing feature selection algorithms, such as info gain, consistency subset, chi-squared, relief, filtered attribute, CFS, filtered subset, and gain ratio. The study highlighted feature selection but provided limited details on PSO parameter configuration and reproducibility. Cherian et al. (27) suggested a weight-optimized neural network for HD detection using hybrid lion and PSO. The dimension reduction issue was solved by the principal component analysis method. The proposed method was implemented in MATLAB 2018a on the Statlog HD dataset and compared the results with other metaheuristic algorithms. This is a strong example of PSO hybridization, but the approach was not validated across multiple datasets. MahaLakshmi et al. (28) proposed an effective HD prediction model using improved PSO and an ensembled classification technique. The model was implemented on two datasets, namely Shahid Rajaei Hospital and UCI Cleveland datasets. In this study, optimal feature subsets were determined by using recursive feature elimination and improved particle swarm optimization (IPSO) algorithms. This study is closely related to ours, but our proposed IPSO incorporates distinct improvements (dynamic inertia and mutation) and a more robust preprocessing pipeline.

Zomorodi-Moghadam et al. (29) suggested a hybrid approach for finding classification rules for the diagnosis of coronary HD. This approach used a binary real PSO technique for determining the velocity of the categorical and numerical particles. In this study, two different feature selection methods based on multi-objective evolutionary search and PSO were employed on the Cleveland HD dataset, and the most important attributes were auto picked by the models. Shahid et al. (30) employed a coronary artery disease diagnosis model using a hybrid PSO-based emotional neural network. They used four different feature selection approaches, such as Fisher, minimum redundancy maximum relevance, relief-F, and weight by SVM, on the Z-Alizadeh Saini dataset to enhance the performance of the proposed model. They obtained better performance measures, which are competitive with the known existing models. Muthukaruppan et al. (31) proposed a hybrid PSO-based fuzzy intelligent system for the diagnosis of coronary artery disease. The simulation results performed on the Cleveland and Hungarian HD datasets were tuned to the fuzzy membership functions by the PSO method. Tang et al. (32) suggested an effective HD classification approach based on a multi-view convolutional neural network with leader and long-tail PSO. This study analyzed the finest details of medical images and built a more appropriate system with maximum accuracy and minimum error. Publicly available datasets from the UCI machine learning repository, particularly the Cleveland and Hungarian datasets, have become benchmarks for developing and validating these intelligent systems. This survey synthesizes recent research (2021–2025) that utilizes these datasets, highlighting methodologies, key findings, identified gaps, and future research directions, as shown in Table 1.

**Table 1:** Summary of comparative literature on heart disease prediction.

**S. No**.	**Reference**	**Methodology**	**Dataset(s)**	**Key findings**	**Gaps identified**	**Limitations**	**Suggested future studies**
1	Zhenya et al. (1)	Hybrid Cost-Sensitive Ensemble	Cleveland, Hungarian, Statlog	Addressed class imbalance; achieved 82.07% (Cleveland) and 79.87% (Hungarian) accuracy	Standard models are sensitive to class distribution	Performance on imbalanced Hungarian dataset was lower	Explore advanced data-level and algorithm-level solutions for severe class imbalance
2	Ozcan et al. (6)	Classification and Regression Tree (CART)	Combined (Cleveland, Statlog, Hungarian, Switzerland, Long Beach)	Achieved 87.25% accuracy by analyzing feature influence	Lack of advanced optimization for feature selection and model hyperparameters	Model may not capture complex, non-linear relationships as well as ensemble methods	Integrate feature selection algorithms and hybridize with ensemble learners
3	Bhatt et al. (18)	XGBoost	Kaggle HD Dataset	Achieved 87.02% accuracy	Did not utilize the standard UCI Cleveland/Hungarian benchmarks for direct comparison	Findings may not be directly comparable to studies on UCI datasets	Benchmark the model on Cleveland and Hungarian datasets to ensure comparability
4	Hera et al. (19)	Multi-Tier Ensemble (MTE) with Random Forest	Cleveland, Statlog	Achieved 88.33% (Cleveland) and 85.19% (Statlog) accuracy	Performance varies across different datasets	The ensemble structure can be computationally expensive	Investigate dynamic ensemble selection based on dataset characteristics
5	Rani et al. (20)	Genetic Algorithm (GA) + RFE + Random Forest	Cleveland	Achieved 86.6% accuracy with effective feature selection	Focused on a single dataset (Cleveland)	Lack of validation on the more challenging Hungarian dataset	Apply and validate the hybrid feature selection method on the Hungarian dataset
6	Koppu et al. (22)	Fitness-Oriented Dragonfly Algorithm (F-DA) + Deep Belief Network	Cleveland, Statlog	Achieved 84.44% (Cleveland) and 86.5% (Statlog) accuracy	Noted very low precision (30.98%) on the Cleveland dataset	Significant class imbalance issue not adequately handled, leading to high FPs	Integrate robust cost-sensitive learning or sampling techniques to improve precision
7	Tama et al. (23)	Two-Tier Classifier + PSO	Cleveland, Hungarian	Achieved high accuracy: 85.71% (Cleveland) and 91.18% (Hungarian)	The two-tier model adds complexity to the training process	The model's decision-making process may be difficult for clinicians to interpret	Develop *post-hoc* explanation techniques to make the model's predictions interpretable
8	Amin et al. (24)	Vote Ensemble (Naïve Bayes + Logistic Regression)	Cleveland	Achieved 87.41% accuracy by combining two simple classifiers	Limited feature engineering and optimization	Simple ensemble may not leverage the full potential of complex, non-linear data	Incorporate feature selection and advanced optimization techniques like PSO
9	Mohan et al. (21)	Hybrid Random Forest with Linear Model (HRFLM)	Cleveland	Achieved high sensitivity (92.8%) and 88.4% accuracy	Model was not tested for generalization on other HD datasets	High sensitivity might come at the cost of a higher false positive rate	Validate the model's specificity and generalizability on the Hungarian and Statlog datasets
10	Proposed Approach (This Study)	PSO + XGBoost (Model 5)	Combined (All five datasets)	Achieved SOTA 91.3% accuracy at 90:10 ratio; high DOR (116.53); validated on Cleveland & Statlog	Previous studies often neglected advanced outlier handling (Median Imputation)	Slightly lower sensitivity than some studies, though still high (88.37%)	Implement advanced feature selection; extend to multiclass classification problems

From the aforementioned literature, it is apparent that most of these previously proposed techniques have utilized various types of feature selection methods, data refining, and optimization techniques in conjunction with several computational models to improve HD detection results. Although previous studies have achieved good results in heart disease prediction, there is still room for improvement. One of the key areas where improvement is needed is in the data preprocessing (33), feature selection (34), and outlier dealing methods (35). The data used to train heart prediction models is often noisy and incomplete. This can lead to inaccurate predictions, as the models may not be able to learn the true relationships between the features and the outcome. Effective data preprocessing methods can help to clean up the data and remove noise, which can improve the accuracy of the models. Feature selection is another important area where improvement is needed. This is desired because not all features are equally important, and including irrelevant features can actually reduce the accuracy of the models. Effective feature selection methods can help to identify the most important features, which can enhance the accuracy of the models. From the above-mentioned literature (1, 6, 18–32), mostly articles have proposed different data preprocessing techniques, optimization techniques, and several ML models, but none of them have given attention to the outliers, which is also necessary for better training any ML model. However, several critical gaps persist:

- Lack of robust preprocessing: many studies overlook the systematic handling of outliers, which can significantly skew model performance. The combined use of padding interpolation, median imputation, and RobustScalar as a unified preprocessing suite is novel in the context of HD prediction.
- Standard PSO limitations: while PSO is popularly used, its tendency for premature convergence is a known issue. An improved PSO variant with mechanisms to escape local optima is needed for more reliable feature selection and model optimization.
- Insufficient statistical validation: many studies report performance metrics but lack statistical significance tests to substantiate that improvements are not due to random chance.
- Limited ablation studies: the individual contribution of preprocessing steps and the optimization algorithm is often not quantified.
- Computational efficiency: discussions on the computational cost and scalability of the proposed models are often missing.

Motivated by the facts mentioned above, in this study, the authors proposed five computational methods for earlier and accurate HD classification that utilizes padding interpolation for missing values (36), median imputation for outliers (37), robust scaling technique (38), several ML models for classification (39), and PSO for optimization of the models (28, 40). The key contributions of this research study can be summarized as follows:

- To the best of my knowledge, padding interpolation, median imputation, and RobustScalar have not yet been applied for filling missing values, eliminating outliers, and scaling whole features in the literature of HD prediction. Hence, this study is the first to introduce their potential use for data filtering, synthesis, scaling, and exploratory data analysis.
- The authors presented a thorough analysis of five heterogeneous classifier structures on different training sets to examine the significant impact on their performance for HD prediction.
- The weight of each hyperparameter of each classifier is optimized by an improved PSO technique for every training set and the proposed best hybrid diagnostic Model 5 (IPSO+XGBoost Classifier) with promising and competitive results.
- The proposed model was tested and validated on two different datasets, namely Cleveland and Statlog, and concluded that the optimized model also achieved consistent performance on these datasets at every training set.
- Compared with existing studies, the proposed hybrid model attained excellent classification results in less computation time and complexity, which implies that it could be a useful tool for clinicians to help them make more accurate and informed decisions about patient care.

The rest of the research study is structured as follows. **Section 2** covers the materials and methodology used in this study. **Section 3** describes machine learning model selection for classification, and **Section 4** represents the particle swarm optimization technique to optimize the weights of several machine learning models. **Section 5** describes the experimental results along with the discussion. Finally, **Section 6** concludes the research study and suggests some future scopes.

## 2 Material and methodology

### 2.1 Dataset acquisition

**Figure 1:**
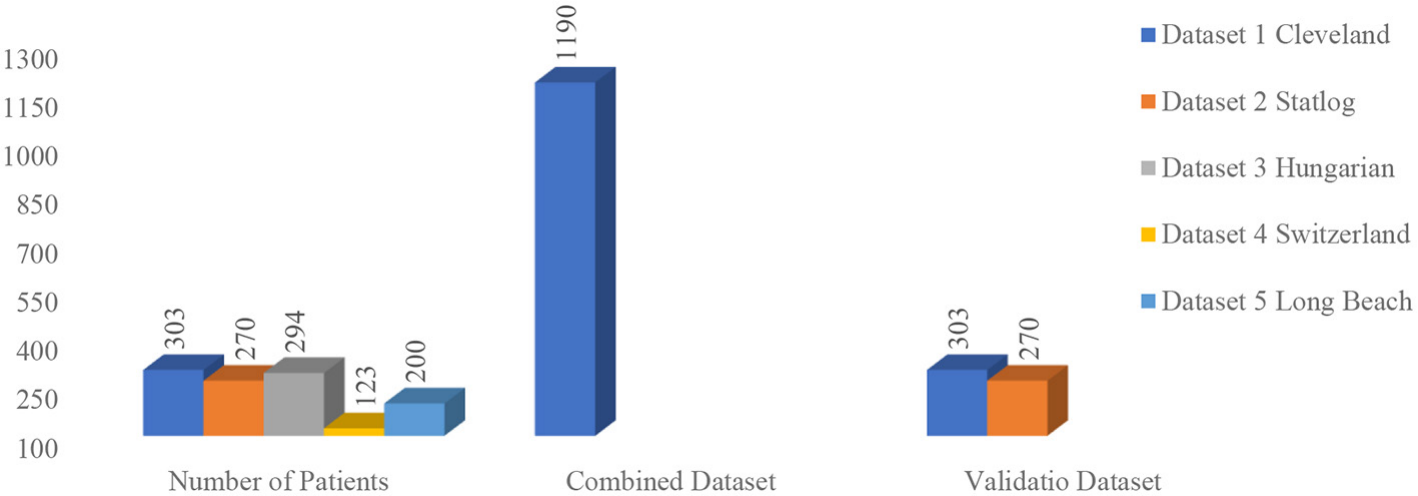
Dataset profile.

This research study utilized five open-source datasets [Cleveland (303), Statlog (270), Hungarian (294), Switzerland (123), and Long Beach (200)] that are readily accessible from the UCI ML Repository (40, 41) as shown in Figure 1. All the dataset includes 14 common features such as age, gender, types of chest pain, resting blood pressure, fasting blood sugar, serum cholesterol, resting electrocardiographic, maximum heart rate achieved during exercise, exercise-induced angina, ST depression induced by exercise relative to rest, number of major vessels colored by fluoroscopy, slope of the peak exercise ST segment, and thalassemia that have been collected to assess the risk factors associated with HD. These features encompass demographic information, medical history, and clinical measurements. All five datasets were merged, resulting in a dataset comprising data from 1,190 patients with 14 features. It contains 52.86% data of people with HD, and the remaining 47.14% have no HD. This dataset comprises 24% of female data and 76% of male data. The detailed description of the dataset has been succinctly summarized in Table 2.

**Table 2:** Description of the heart disease dataset.

**S. No**	**Parameters**	**Description**	**Type**	**Data range**
1	age	Years	Integer	[28 to 77]
2	sex	Male or female	Integer	•0 = Female •1 = Male
3	cp	Type of chest pain	Integer	•0 = Typical angina •1 = Atypical angina •2 = Non-angina pain •3 = Asymptomatic
4	thestbps	Resting blood pressure	Integer	[80–200 mm/Hg]
5	chol	Cholesterol	Integer	[85–603 mm/dl]
6	Restecg	Resting electrographic	Integer	•0 = normal •1 = having ST_T wave abnormal •2 = left ventricular hypertrophy
7	fbs	Fasting blood sugar	Integer	•1 ≥ 120 mg/dl •0 ≤ 120 mg/dl
8	thalach	Maximum heart rate	Integer	[60–202 bpm]
9	exang	Exercise-induced angina	Integer	•0 = No •1 = Yes
10	oldpeak	ST depression induced by the workout relative to rest	Float	Continuous value [-2.6 to 6.2]
11	slope	Slope of the peak workout ST segment	Integer	•0 = Unsloping •1 = Flat •2 = Down sloping
12	ca	The number of the main vessels	Integer	[0–3]
13	thal	Thalassemia	Integer	•0 = normal •1 = fixed defect •2 = reversible defect
14	target	A person who has suffered from heart disease or not	Integer	•1 = Yes (present) •0 = No (absent)

### 2.2 Data preprocessing and feature engineering

Data preprocessing refers to the set of techniques and procedures employed on raw data before it is used for further analysis or modeling purposes. It involves transforming, scaling, encoding, cleaning, and organizing the data to enhance its quality, consistency, and usability. In this research study, the authors used some preprocessing steps that are explained in the next subsections (33, 34, 42–44).

#### 2.2.1 Padding interpolation and correlation

The missing/null values can be handled by some basic methods, such as by removing or replacing them with the average value, median value, and mode value, but these methods are not the best fit for every dataset. Another advanced technique for handling missing/null values is the “Interpolation technique” (45). Interpolation is appropriate when the researchers are dealing with datasets that have missing values, irregular sampling intervals, or noisy data points. It is mostly used to impute the missing values in the data frame while preprocessing. Although there are various Interpolation methods available in the literature, the most common techniques are linear interpolation, polynomial interpolation, and padding interpolation (36). In this research study, the authors have handled the missing values through the “Padding Interpolation” method. In this method, missing values are filled with the preceding value of the dataset. In case the value is also missing in the preceding row, then this method will not work. In this study, there is no missing value in the preceding row of the dataset; hence padding interpolation is suitable in this scenario (46–48). The pseudocode of padding interpolation is mentioned in Table 3. Correlation is a statistical measure that determines the relationship between two or more variables. It can provide insights into how changes in one variable may affect another variable. It is often used to identify patterns, dependencies, or associations between features of the dataset. The most frequently used correlation is the Pearson correlation, which measures the linear relationship between two continuous variables. It ranges from −1 (representing a perfect negative linear relationship) to +1 (indicating a perfect positive linear relationship), with 0 indicating no linear relationship. Other correlations, such as Spearman's rank and Kendall's rank, are used when dealing with non-linear relationships or ordinal variables. In this study, Pearson's correlation method has been used, as stated in Equation 1 (39, 49).


$$\begin{array}{l} r=\frac{\sum \left(X-\;\bar{X}\right)\left(Y-\;\bar{Y}\right)}{\sqrt{\sum {\left(X-\;\bar{X}\right)}^{2}{\left(Y-\;\bar{y}\right)}^{2}}} \end{array}$$ (1)

**Table 3:** Pseudocode of padding interpolation.

**Padding Interpolation pseudocode:**
1. **Input:** Dataset with missing values (data) 2. **For** each feature (column) in the dataset: 3. **For** each data point in the feature: 4. **If** the data point is missing: 5. Initialize variables for left_neighbor and right_neighbor as **None** 6. Set left_index and right_index as the index of the previous and next non-missing data, respectively. 7. **If** left_index is not **None**: - Set left_neighbor as the value of the data point at left_index. 8. **If** right_index is not **None**: - Set right_neighbor as the value of the data point at right_index. 9. **If** both left_neighbor and right_neighbor is not **None**: - Perform Interpolation to estimate the missing value based on the neighboring values. - Replace the missing value with the estimated value. 10. **Output:** Dataset with missing values filled using Padding Interpolation.

where ∑ denotes the summation, *X* and *Y* are the variables for calculating the correlation, $\bar{X}$ and $\bar{Y}$ denotes the mean of *X* and *Y*, respectively.

#### 2.2.2 Outliers imputation

Outliers in a dataset refer to data points that deviate significantly from the rest of the observations. They are data points that are located far away (either extremely high or low values) from the majority of the data and do not follow the general pattern or distribution. It can occur due to various reasons, including data entry mistakes, measurement errors, natural variations, or rare events. They can have a significant effect on data analysis and modeling as they can distort statistical measures, affect the estimation of parameters, and finally influence the overall analysis results. There are several ways to find the outliers, i.e., scatter plot, box plot, *z*-score, and interquartile range (IQR) (50). In this study, the authors used the inter-quartile range (IQR) approach to identify outliers in the dataset. It involves calculating the range between the first quartile (Q1) and the third quartile (Q3) of the dataset. It is a number that shows how scattered the dataset is and indicates the range score. Q1 represents the first quartile of the dataset, i.e., 25% of the data lies between the minimum and Q1. Similarly, Q3 indicates that 75% of the data lies between the minimum and Q3. Afterward, the decision boundary is defined by using Equations 2, 3 (51, 52), and any data that falls outside this range is considered an outlier.


$$\begin{array}{l} Lower\;bound=\left(Q1-1.5*IQR\right) \end{array}$$ (2)

$$\begin{array}{l} Upper\;bound=\left(Q3+1.5*IQR\right) \end{array}$$ (3)

Afterward, once outliers are detected, there are several approaches to handling them, such as removing them, transforming them, and treating them as separate classes, depending on the nature of the data. In this study, the authors used the median imputation method to handle the outliers, whose pseudocode is given in Table 4 (37).

**Table 4:** PseudoCode of median imputation.

**Median Imputation pseudocode:**
**Input:** Dataset with outlier values (data) 1. Calculate the median value of each feature in the dataset. 2. **For** each feature in the dataset: 2.1. Identify outliers using an outlier detection method (e.g., IQR method). 2.2. **For** each outlier value in the feature: - Replace the outlier value with the median value of the feature. 3. **Return** the dataset with the Median Imputed values.

### 2.3 Robust scaling

Scaling refers to the process of transforming the numerical features of this dataset into a specific scale or range. It is a preprocessing step that ensures that all the values of different features are normalized on a particular scale. Several scaling techniques, such as standardization, normalization, and robust scaling, are available in the literature. In this study, the authors have used an advanced scaling method, “RobustScalar,” to scale the whole dataset in a particular range. In this scaling method, the values are scaled based on the median and interquartile range (IQR) instead of the mean and standard deviation. This technique ensures that the scaled values maintain their relative ordering and distribution while maintaining the sacredity of the dataset. This scaling method is particularly useful when the dataset contains significant outliers or when the assumption of a normal distribution is violated. The mathematical representation of robust scaling is (17, 38):


$$\begin{array}{l} Robust\;Scaled\;Value=\frac{Original\;value-Input^{\prime }s\;Median}{Input^{\prime }s\;IQR} \end{array}$$ (4)

## 3 Machine learning model selection

There are several ML models such as logistic regression (53–60), linear discriminant analysis (LDA) (61, 62), support vector classifier (63), Gaussian Naïve Bayes (13), extreme gradient boosting (15, 64–67), decision tree (51), etc., are available for prediction and classification problems. Over the last two decades, these ML models have been widely used in the medical sector and have been briefly explained in the next subsections (39).

### 3.1 Logistic regression

Logistic regression (LR) is a standard classification technique employed for regression as well as classification problems (59). Pierre Francois Verhulst, a French mathematician, developed the logistic equation in the 19th century to explain how human populations grow. (60). A review of the literature published between 2000 and 2018 found that 43 publications (six textbooks and 37 articles) were focused on logistic regression (LR), demonstrating its importance as a leading intelligent system. It helps in discovering the probability of an event (e.g., yes or no) and its probability ranges between 0 and 1. The activation function of LR is either a Sigmoid or a Logistic function, which is given in Equation 5 (53–58).


$$\begin{array}{l} P=\frac{1}{1+e^{-\left(b_{0}x_{0}+b_{1}x_{1}+\ldots +b_{n}x_{n}\right)}} \end{array}$$ (5)

Here *P* is the binary response variable, _*x*_0_, *x*1_, *x*_2_…*x*_*n*_ stand for instances, and _*b*_0_, *b*1_, *b*_2_…*b*_*n*_ are the logistic coefficients.

### 3.2 Linear discriminant analysis

Linear discriminant analysis (LDA) was first introduced by Fisher (61, 62) and is basically a supervised machine learning algorithm utilized for dimensionality reduction and classification problems. Its objective is to find a linear combination of attributes that maximizes the separation between two or more classes in the dataset. It assumes that the data is normally distributed within each class (Gaussian distribution), and these classes have identical covariance matrices. It transforms the original features into a lower-dimensional subspace. Fisher's two-class separation is defined as:


$$\begin{array}{l} S=\frac{{\left(\vec{\omega }\left({\vec{\mu }}_{1}-{\vec{\mu }}_{0}\right)\right)}^{2}}{{\vec{\omega }}^{T}\left(\sum_{0}+\sum_{1}\right)\vec{\omega }} \end{array}$$ (6)

where ${\vec{\mu }}_{0},{\vec{\mu }}_{1}$are means of two classes, $\underset{0}{\sum },\underset{1}{\sum }$ are covariances of two classes, ${\vec{\omega }}^{T}$ linear combination of features, and $\vec{\omega }$ is an eigenvector.

### 3.3 Support vector classifier

Support vector classifier (SVC) is a supervised ML algorithm known for its ability to handle complex data and find the finest decision boundaries. For classification problems, the core purpose of SVC is to find a hyperplane (depending on the features present in the dataset), which separates data points from different classes in a more appropriate way. The hyperplane is defined as a multidimensional surface that maximizes the boundary between the two classes, thereby improving the ability of the model. SVC can handle a non-linear dataset by mapping the input attributes into a higher-dimensional space using kernel functions. SVC can also be used for regression tasks, where the goal is to fit a hyperplane that captures as many data points as possible within a stated margin. The mathematical expression of SVC is (63):


$$\begin{array}{l} y\left(x\right)=sign\left[\overset{N}{\underset{i=1}{\sum }}\alpha_{i}y_{i}K\left(x_{i},x\right)+b\right] \end{array}$$ (7)

where *y*(*x*) is the objective function, α_*i*_ denotes the positive real constants, *b* is a real constant, *K*(*x*_*i*_, *x*) is kernel function, α_*i*_ is Lagrange multiplier, *x*_*i*_ is data points, and *y*_*i*_ is the label of data points.

### 3.4 Gaussian Naïve Bayes

Gaussian Naïve Bayes (GNB) is a variant of the Naïve Bayes algorithm that assumes that the features in the dataset are continuous and follow a Gaussian (normal) distribution. It is a probabilistic supervised ML classifier that uses Bayes' theorem to make predictions based on the probability of an instance belonging to a particular class. It assumes that the presence of a specific feature in a class is independent of any other feature. GNB is compatible with large datasets and can perform well even in complex scenarios. Bayes' theorem is expressed in mathematical form and is given in Equation 8 (13).


$$\begin{array}{l} P\left(\frac{\alpha }{\beta }\right)=\frac{P\left(\frac{\beta }{\alpha }\right)\times P\left(\alpha \right)}{P\left(\beta \right)} \end{array}$$ (8)

where α is the class in a dataset, β is the features present in a dataset, $P\left(\frac{\alpha }{\beta }\right)$ indicates the posterior probability, $P\left(\frac{\beta }{\alpha }\right)$ represents the likelihood, which is the probability of the predictor, *P*(α) represents the class prior probability, and *P*(β) denotes the predictor's prior probability.

### 3.5 Extreme gradient boosting classifier

Extreme gradient boosting classifier, also known as XGBoost Classifier, is a supervised ML algorithm. It is an advanced variant of the gradient boosting machine (GBM) and was proposed by Friedman (65). This method combines multiple weak predictive models (typically decision trees) to build a strong predictive model, without disturbing the previous classifier, and is repeated until the desired accuracy is attained. The final outcome is determined by the ensemble of all the decision trees' outputs. It supports distributed computing and can manage large datasets with millions of samples and high-dimensional feature spaces. The optimized mathematical objective function of XGBoost classifier is shown in Equation 9 (15, 64–67).


$$\begin{array}{l} Obj^{\left(t\right)}=\overset{n}{\underset{i=1}{\sum }}\left[L\left(y_{i},{ŷ}_{i}^{\left(t-1\right)}\right)+g_{i}f_{t}\left(x_{i}\right)+\frac{1}{2}h_{i}f_{t}^{2}\left(x_{i}\right)\right]\\ \;+R\left(f_{t}\right)+constant \end{array}$$ (9)

where *L* stands for the loss function, ŷ_*i*_ stands for the predicted (*i*^*th*^) label, *y*_*i*_ denotes the actual (*i*^*th*^) label, *R*(*f*_*t*_) stands for penalizing the complexity of the training function, *n* is the number of samples, *t* stands for *t*^*th*^ observation, *f*_*t*_ is adding to minimize the objective function error, *g*_*i*_ is the first-order gradient function and *h*_*i*_ is second-order gradient statistics.

## 4 Improved particle swarm optimization (IPSO)

Particle swarm optimization (PSO) is a population-based metaheuristic optimization approach inspired by the collective behavior of bird flocking or fish schooling, developed by Eberhart and Kennedy in 1995. The particles in the swarm are like birds or fish, which move together through the search space and share information about their best positions. This allows the algorithm to find the best solutions for the problems, which are difficult to solve by other methods. In PSO, a population of “n” particles is used to represent potential solutions to the problem. Each particle has a position and velocity, which are updated over time according to three factors such as inertia weight, local best (personal experience velocity), and global best (entire swarm velocity). The PSO algorithm starts with a random population of “n” particles. The particles then move through the search space, and their positions and velocities are updated according to the above three factors. The algorithm terminates when a stopping criterion is met, such as a maximum number of iterations or a minimum fitness value. PSO is a simple and efficient algorithm that can be used to solve a variety of optimization tasks, including continuous optimization tasks, discrete optimization tasks, and combinatorial optimization tasks. It is especially well-suited for problems with continuous search spaces (28, 40). The fundamental steps of the PSO algorithm are given in Table 5.

**Table 5:** Pseudocode of the Particle Swarm Optimization Algorithm.

**Particle Swarm Optimization pseudocode:**
Step 1. Assign c1, c2, and w from control parameters. Step 2. Create starting particles. Step 3. Calculate fitness function value for each particle in the swarm. Step 4. REPEAT the process. Step 5. Compare fitness value and change with the best value. Step 6. Evaluate global best velocity and position. Step 7. Update the best velocity and location. Step 8. RETURN global best position as the optimal solution

However, the standard PSO algorithm is often plagued by the issue of premature convergence, where the swarm gets trapped in a local optimum before finding the global best solution. To overcome this limitation and enhance the optimization performance for the heart disease prediction task, the authors proposed an improved PSO (IPSO) algorithm. The improvements are twofold:

- Dynamic Inertia weight (w): instead of a fixed value, the inertia weight is dynamically updated during the optimization process. It starts with a higher value (*W*_max_) to promote global exploration and linearly decreases to a lower value (*W*_min_) to facilitate local exploitation in later iterations. This is calculated as:

$$\begin{array}{l} W=\frac{W_{\max}-\left(\left(W_{\max}-W_{\min}\right)\times Current_{iteration}\right)}{Max_{iteration}} \end{array}$$ (10)

- Mutation operator: to further prevent premature convergence, a Gaussian mutation is introduced with a certain probability (*P*_*mutation*_). This operator randomly perturbs the position of a particle, allowing it to escape local optima.

$$\begin{array}{l} particle\left[position\right]\;=\;particle\left[position\right]\;+\;N\left(0,\;\sigma \right) \end{array}$$ (11)

where *N*(0, σ) is a random number drawn from a Gaussian distribution with mean 0 and a small standard deviation σ.

The mathematical formulation of the IPSO is as follows: each particle i has a position vector X_i_ = (x_i1_, x_i2_, ..., x_iD_) and a velocity vector V_i_ = (v_i1_, v_i2_, ..., v_iD_) in a D-dimensional search space. The particles update their velocity and position using Equations 12, 13, respectively:


$$\begin{array}{l} \;Vi\left(t+1\right)\;=\;w\left(t\right)\;*Vi\left(t\right)\;+\;c1\;*r1\\ {}*\left(Pbest<uscore>i\left(t\right)\;-\;Xi\left(t\right)\right)\;+\;c2\;*r2\;*\left(Gbest\left(t\right)\;-\;Xi\left(t\right)\right) \end{array}$$ (12)

$$\begin{array}{l} Xi\left(t+1\right)\;=\;Xi\left(t\right)\;+\;Vi\left(t+1\right) \end{array}$$ (13)

where

- w(t) is the dynamic inertia weight from Equation 10.
- c1 and c2 are the cognitive and social acceleration coefficients, respectively.
- r1 and r2 are random numbers uniformly distributed in [0, 1].
- Pbest_i(t) is the best position particle i has achieved so far.
- Gbest(t) is the best position found by any particle in the swarm so far.

The objective function (fitness function) for the IPSO is defined as the maximization of the classifier's performance. This method uses the F1-score as it provides a balanced measure between precision and recall. The pseudocode of the proposed IPSO algorithm is presented in Table 6.


$$\begin{array}{l} \;Fitness\;=\;F1-Score\\ =\;2\;*\left(Precision\;*Recall\right)/\left(Precision\;+\;Recall\right) \end{array}$$ (14)

**Table 6:** Pseudocode of the improved particle swarm optimization (IPSO) algorithm.

**1. Input:** Population size (*n*), max_iterations_, c1, c2, w_max_, w_min_, P_mutation_, σ. 2. **Initialize** a population of particles with random positions and velocities. 3. **For** each particle, evaluate the desired fitness function (F1-Score of the classifier with the current particle's position as hyperparameters/feature mask). 4. **Initialize** P_best_ of each particle to its current position. Initialize G_best_ to the position of the particle with the highest fitness. 5. **For** iteration = 1 to max_iterations_: 1. Calculate dynamic inertia weight: w = w_max_ - ((w_max_ - w_min_) ^*^ iteration)/max_iterations_ 2. **For** each particle in the swarm: 1. Update velocity using Equation 13. 2. Update position using Equation 14. 3. **If** (rand() <P_mutation_): 1. Apply Gaussian mutation to the particle's position using Equation 12. 4. Evaluate the particle's fitness. 5. **If** the current fitness is better than its P_best_ fitness: 1. Update P_best_ to the current position. 6. **If** the current fitness is better than the G_best_ fitness: 1. Update G_best_ to the current particle's position. 6. **Output**: G_best_ (The optimal solution: selected feature subset and/or hyperparameter set).

### 4.1 Proposed hybrid ML models based on improved particle swarm optimization

Although the basic ML models can achieve the desired output, there is still a need for hyperparameter optimization to enhance the model's performance, robustness, flexibility, and interpretability. After a rigorous literature survey, the authors have analyzed that the traditional or basic hyperparameter tuning, such as manual search, grid search, random SearchCV, and Bayesian search, has been inefficient for high-dimensional hyperparameter spaces, easily stuck in local optima, and requires a specific set of parameter values. But on the other hand, improved particle swarm optimization (IPSO) is a more efficient metaheuristic optimization technique for high-dimensional datasets and is less likely to get stuck in local optima. In this research study, the authors hybridized and optimized all five ML models mentioned in **Section 3** with PSO to optimize the weights of the models. The proposed five hybridized models are Model 1 (PSO+LR), Model 2 (PSO+LDA), Model 3 (PSO+SVC), Model 4 (PSO+GNB), and Model 5 (PSO+XGBoost Classifier). The PSO technique is inspired by the collective behavior of bird flocking, which has a strong global search capability to choose the best particle value in a swarm or population. The proposed models first initialize the PSO coefficients, such as the cognitive coefficient (c1), social coefficient (c2), and inertia weight (w) (27, 28, 68). In the next step, an initial population of particles was generated, where each particle denotes a set of all ML model coefficients. The position and velocities of the particles within the swarm are set randomly to identify the global best position with the fitness value among the population. Finally, all the ML models were trained with newly generated swarm values and evaluated based on performance metrics, that is, accuracy, sensitivity, specificity, F1 score, and others. If the fitness value is better than the particle's previous best value, then the local best and global best fitness value (54) are updated. The advanced hybrid pseudocode of all the optimized models is given in Table 7.

**Table 7:** Advanced hybrid pseudocode of the optimized heterogeneous models.

**Advanced pseudocode for all hybrid optimized Model 1, 2, 3, 4, and 5**
1. **Set** population size (*n*), maximum number of iterations (max_iter), and search space boundaries. 2. **Set** IPSO coefficients: cognitive coefficient (c1), social coefficient (c2), and inertia weight (w). 3. Generate an initial population of particles, where each particle represents a set of LR/LDA/SVM/GNB/XGBoost model coefficients. 4. Randomly initialize the positions and velocities of the particles within the search space. 5. Define **global** best position as the position with the best fitness value among the population. 6. **For each** iteration in 1 to max_iter: 7. **For each** particle in the population: 8. Calculate cognitive component: c1 ^*^ random() ^*^ (pbest - current_position) 9. Calculate social component: c2 ^*^ random() ^*^ (gbest - current_position) 10. Update velocity: w ^*^ current_velocity + cognitive_component + social_component 11. Update position: current_position + current_velocity 12. **Set** LR/LDA/SVM/GNB/XGBoost hyperparameters. 13. **Fit** LR/LDA/SVM/GNB/XGBoost model using training data and corresponding labels. 14. Calculate fitness metrics (e.g., accuracy, precision etc.) on a validation set using the trained LR/LDA/SVM/GNB/XGBoost model. 15. **If** fitness value is better than particle's previous best, update pbest and fitness value. 16. **If** fitness value is better than gbest, update gbest. 17. **End for** 18. **Return** the particle with the global best fitness value.

The parameter settings for the IPSO algorithm used across all experiments are detailed in Table 8. These values were determined empirically through pilot studies to balance convergence speed and solution quality.

**Table 8:** Parameter settings for the improved PSO (IPSO).

**Parameter**	**Value**	**Description**
Population size (*n*)	50	Number of particles in the swarm
Maximum iterations	100	Stopping criterion
Cognitive coefficient (c1)	2.0	Influences the particle's movement toward its personal best
Social coefficient (c2)	2.0	Influences the particle's movement toward the global best
Inertia weight (w_max_)	0.9	Starting value for dynamic inertia
Inertia weight (w_min_)	0.4	Final value for dynamic inertia
Mutation probability (P_mutation_)	0.1	Probability of applying Gaussian mutation
Mutation Std. Dev. (σ)	0.1	Standard deviation for Gaussian mutation

### 4.2 Performance evaluation of the ML models

The performance evaluation of ML models involves assessing the model's effectiveness, quality, and reliability, and measuring its ability to make accurate classifications. It provides insights into the model's accuracy, precision, F1-score, recall, and other aspects of its performance, based on the confusion matrix or error matrix of the model. The confusion matrix includes true positives (TP), true negatives (TN), false positives (FP), and false negatives (FN) for each class. The choice of performance evaluation metrics depends on the problem type, such as classification, regression, and clustering. The research problem in this study is of classification type, hence all the ML models were compared according to the criteria of accuracy, balanced accuracy, sensitivity, precision, f1 score, specificity, negative predictive value (NPV), markedness (MK), bookmaker informedness (BM), Matthews correlation coefficient (MCC), false negative rate (FNR), false positive rate (FPR), false discovery rate (FDR), false omission rate (FOR), positive likelihood ratio (LR+), negative likelihood ratio (LR–), and diagnosis odds ratio (DOR) (54, 69–71), as demonstrated in Table 9. Furthermore, to validate the statistical significance of the results, the authors employed a paired *t*-test. The authors compared the performance (e.g., accuracy) of the proposed model against baseline models across multiple runs or different data splits. A *p*-value of less than 0.05 was considered statistically significant.

**Table 9:** Evaluation metrics for the proposed approach.

**S. No**.	**Performance evaluation metrics**	**Formula**	**S. No**.	**Performance evaluation metrics**	**Formula**
1	Accuracy	$\frac{TN\;+\;TP}{TP\;+\;TN\;+\;FP\;+\;FN\;}$	2	Sensitivity (Recall/TPR)	$\frac{TP\;}{FN\;+\;TP\;}$
3	Specificity (TNR)	$\frac{TN}{FP\;+\;TN\;}$	4	Precision (PPV)	$\frac{TP}{FP+\;TP\;}$
5	F1 Score	$\frac{2TP}{\left(FP\;+\;2TP\;+\;FN\right)}$	6	NPV	$\frac{TN}{FN+\;TN\;}$
7	MCC	$\frac{\left(TN\times TP\right)-\left(FP\times FN\right)}{\sqrt{\left(TN+FN\right)\left(TP+FP\right)\left(TN+FP\right)\left(FN+TP\right)}}$	8	BA	$\frac{TPR+TNR}{2}$
9	LR+	$\frac{TPR}{FPR}$	10	LR–	$\frac{FNR}{TNR}$
11	DOR	$\frac{LR+}{LR-}$			

TP, true positive; TN, true negative; FP, false positive; FN, false negative; TPR, true positive rate; TNR, true negative rate; PPV, positive predicted value; NPV, negative predicted value; MCC, Matthews correlation coefficient; BA, balanced accuracy; LR+, positive likelihood ratio; LR–, Negative likelihood ratio; DOR, diagnostic odds ratio.

## 5 Experimental results and discussion

Five open-source datasets (Cleveland, Statlog, Hungarian, Switzerland, and Long Beach) (40, 41) readily accessible from the UCI ML Repository, have been used in this research study. All five datasets were merged, resulting in a dataset comprising data from 1,190 patients with 14 features. The dataset is further processed with the machine having; Processor = “Intel(R) Core (TM) i7-10750H CPU @ 2.60 GHz 2.59 GHz”; Window = “10”; RAM = “8 GB”; Python = 3.8.5; Numpy = 1.21.5; Pandas = 1.2.2; Matplotlib = 3.3.2. All the experiments were validated by applying the Stratified K-Fold Cross-Validation (CV) technique, an extension of the regular K-Fold.

### 5.1 Data synthesis, filtering, and normalization

**Figure 2:**
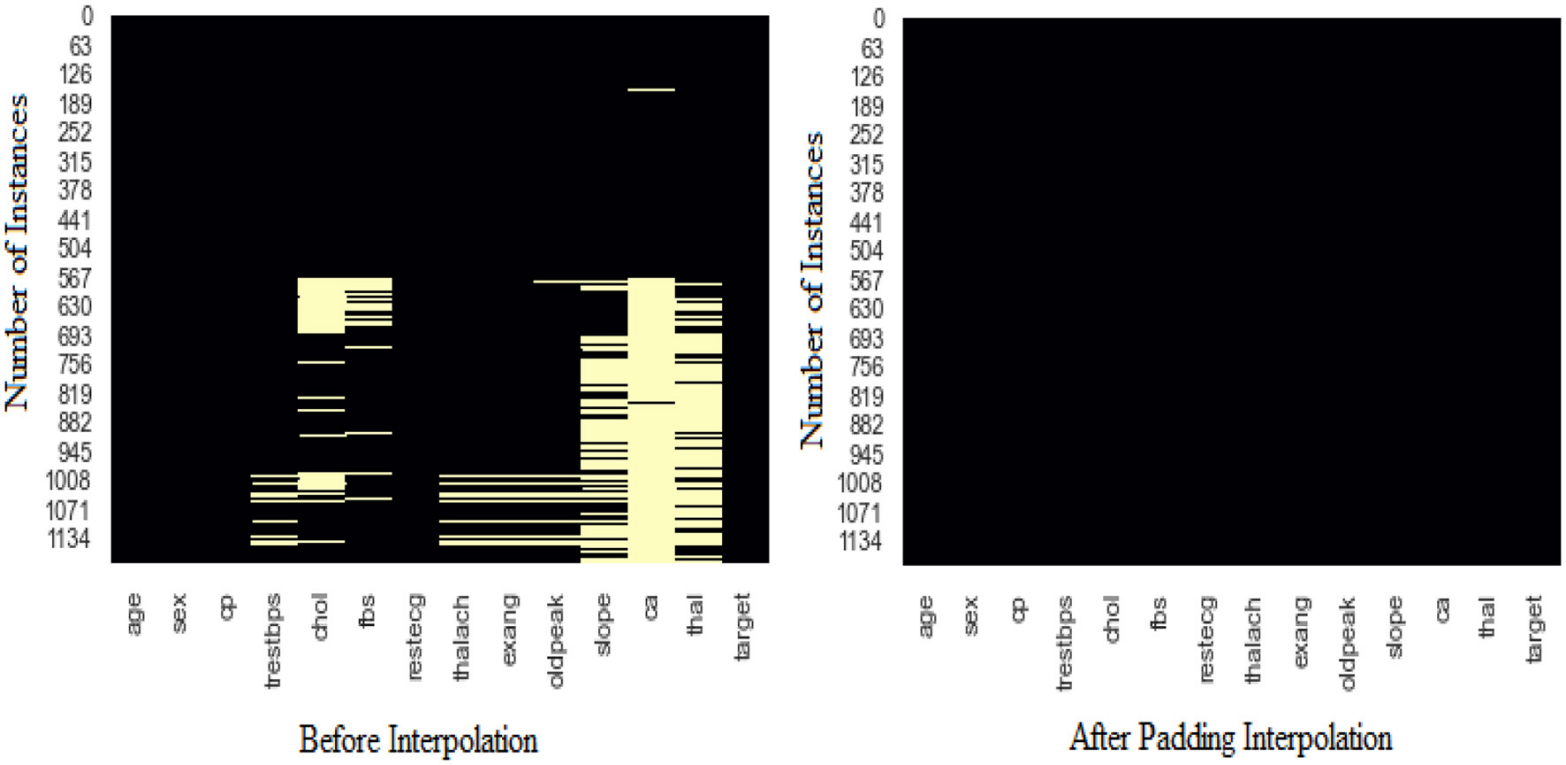
Missing values in the dataset before and after padding interpolation.

The data of 1,190 patients with 14 major attributes comprising 16,660 data points (1,190 × 14) were analyzed thoroughly, and subsequent steps were utilized to address the missing values present in the dataset. In this dataset, a total of 1,924 missing values were identified and addressed. The identified missing values in attributes, such as resting blood pressure, cholesterol, fasting blood sugar, resting electrocardiographic, maximum heart rate, exercise-induced angina, ST depression, slope, number of major vessels, and thalassemia were 60, 202, 90, 2, 55, 55, 62, 308, 611, and 479, respectively. All the missing values were imputed by the advanced technique “Padding Interpolation” with a maximum missing value limit of 611, and the dataset was arranged into the correct format, that is, integer or floating format. The pattern of missing values in the dataset before and after interpolation is portrayed in Figure 2. The dataset contains 52.86% (i.e., 629) of data of people with HD, and the remaining 47.14% (i.e., 561) with normal. The difference between people with HD and without HD is 5.72% which indicates the dataset is almost in a balanced form and does not require any data balancing step.

**Figure 3:**
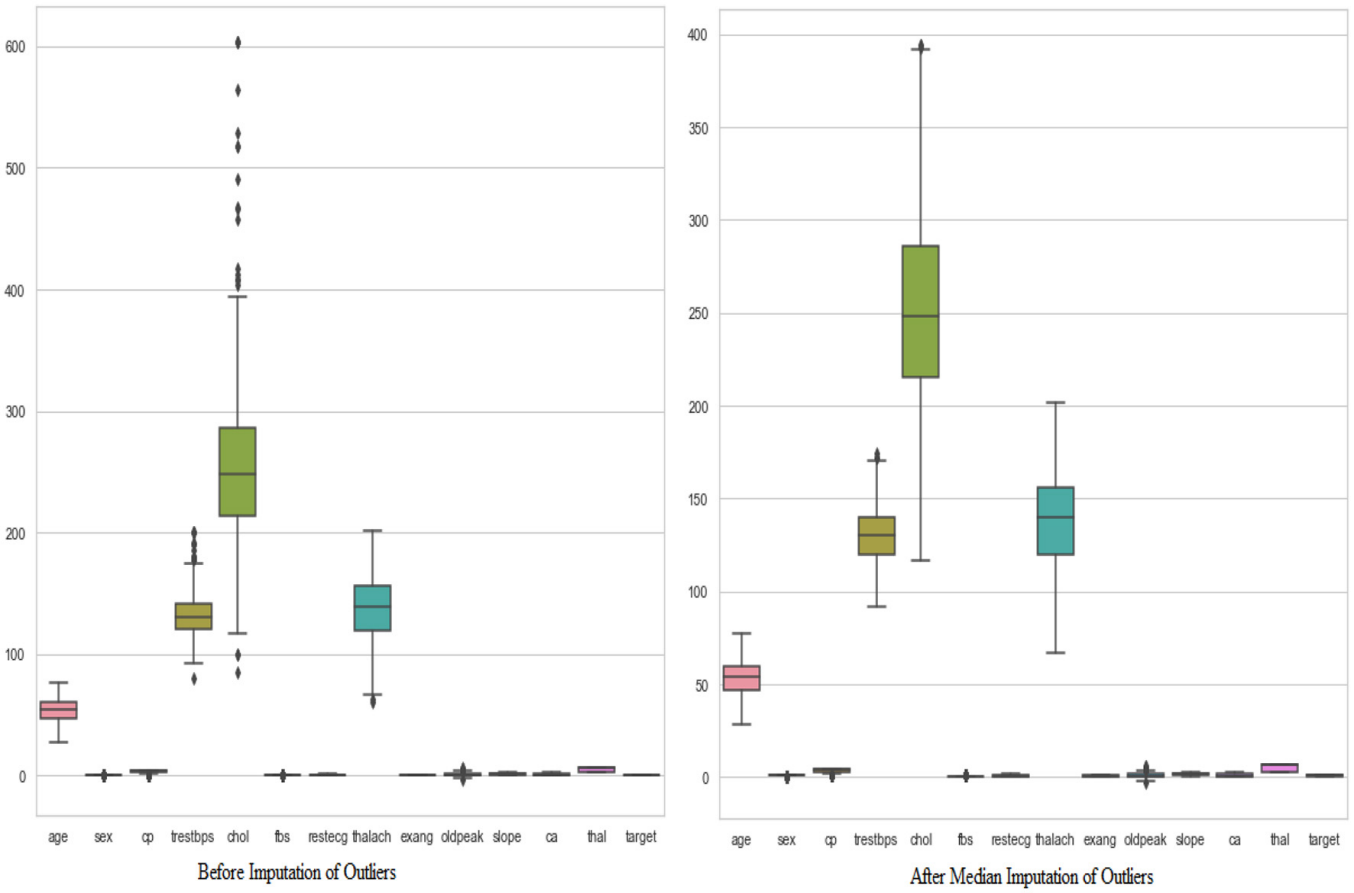
Dataset before and after median imputation of outliers.

**Figure 4:**
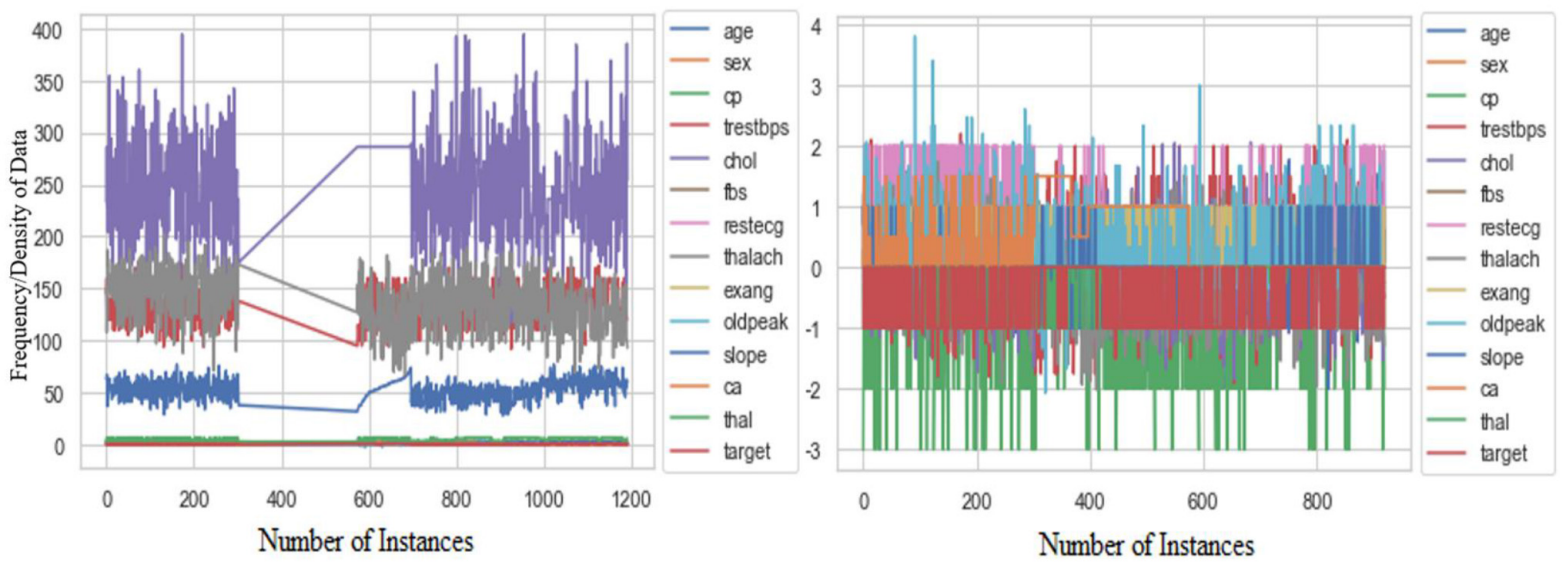
Distribution of the dataset before and after robust scaling.

In the next step, the authors identified 270 (22.69%) duplicate values within the dataset and subsequently removed all these values to prevent biased results. The remaining dataset (920) was further processed to identify the outliers by the interquartile range (IQR) method. All the outliers presented in the dataset were imputed by the median imputation technique to make the data more interpretable. Figure 3 shows the boxplots of key features (e.g., cholesterol and resting blood pressure) before and after median imputation of outliers, demonstrating the effective mitigation of extreme values. Further, the authors used RobustScaler method to scale the whole dataset in a similar range, as illustrated in Figure 4, which shows the distribution of a sample feature (e.g., age) before and after scaling, highlighting how RobustScaler mitigates the effect of any remaining outliers. The crisp and pristine dataset is now ready to be used with different train-test ratios, i.e., 70:30, 80:20, and 90:10.

### 5.2 Experiment 1: default machine learning models training on different train-test ratio

**Figure 5:**
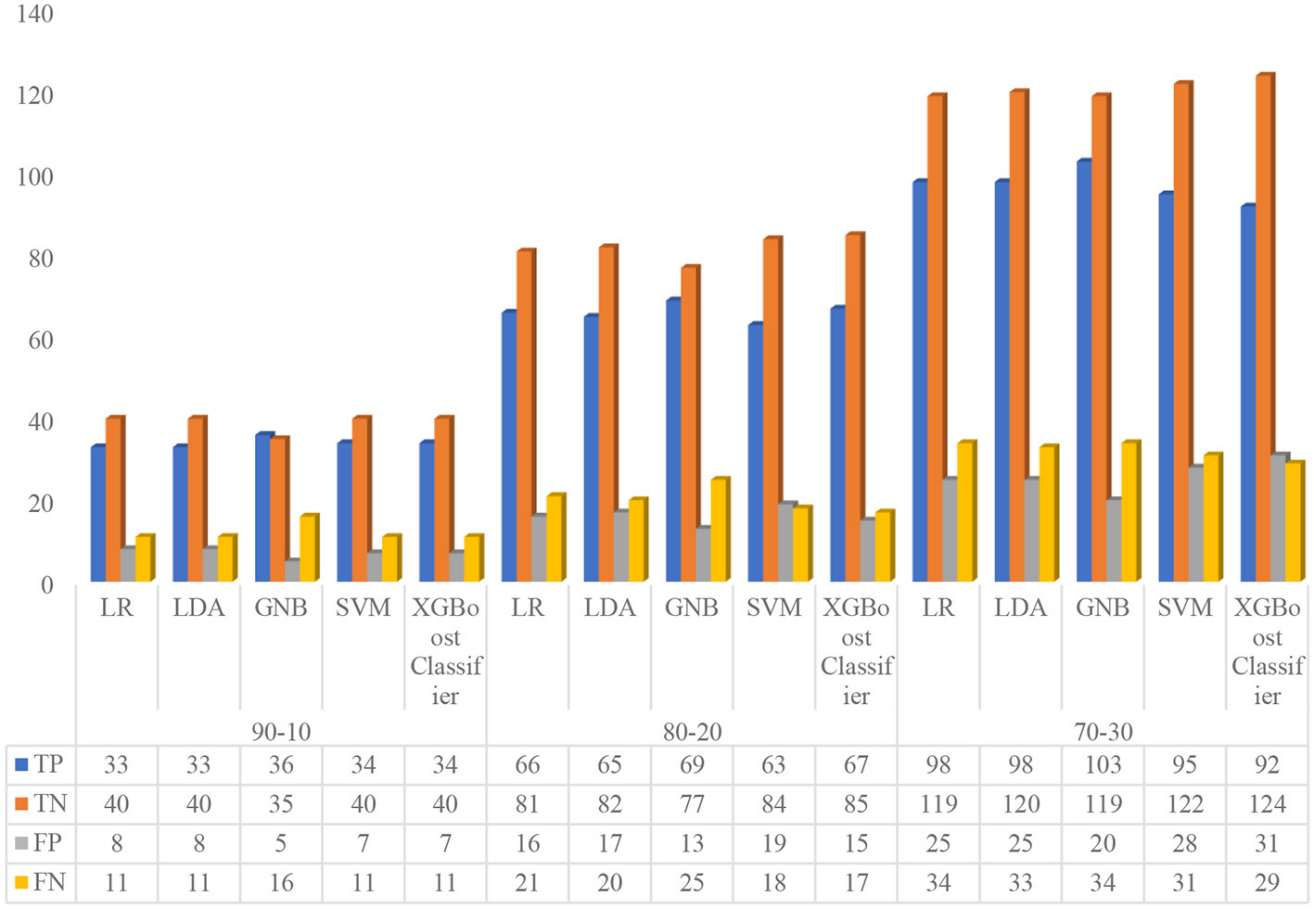
Confusion matrix of five default machine learning models on different training sets.

In this experiment, the authors trained all five default ML models, namely, LR, LDA, GNB, SVM, and XGBoost Classifiers on different train–test ratios (70:30, 80:20, and 90:10) with zero random states. The performance of default models was analyzed based on the confusion matrix and performance metrics, as shown in Figure 5 and Table 10, respectively. From Figure 5, firstly, the confusion matrix for all the default models on 90:10 train-test ratio (i.e., 828 data for training, and 92 data for testing) was analyzed. At this 90:10 ratio, the LR model achieved 33 true positive (TP), 40 true negative (TN), eight false positive (FP), and 11 false negative (FN), respectively; the LDA model attained 33 TP, 40 TN, eight FP, and 11 FN, respectively; the GNB model showed 36 were TP, 35 were TN, five were FP, and 16 were FN, respectively, the SVM model represented 34 were TP, 40 were TN, seven were FP, and 11 were FN, respectively, and the XGBoost Classifier model showed 34 were TP, 40 were TN, 7 were FP, and 11 were FN, respectively. Similarly, the authors analyzed the confusion matrix for all the default models on an 80:20 train–test ratio (i.e., 736 data for training, and 184 data for testing). At this 80:20 ratio, the LR model represented 66 were TP, 81 were TN, 16 were FP, and 21 were FN, respectively; the LDA model attained 65 TP, 82 TN, 17 FP, and 20 FN, respectively; the GNB model showed 69 were TP, 77 were TN, 13 were FP, and 25 were FN, respectively; the SVM model represented 63 were TP, 84 were TN, 19 were FP, and 18 were FN, respectively; and the XGBoost Classifier model obtained 67 were TP, 85 were TN, 15 were FP, and 17 were FN, respectively. Finally, the authors analyzed the confusion matrix for all the default models on 70:30 train–test ratio (i.e., 644 data for training and 276 data for testing). At this 70:30 ratio, the LR model achieved 98 TP, 119 TN, 25 FP, and 34 FN, respectively; the LDA model attained 98 TP, 120 TN, 25 FP, and 33 FN, respectively; the GNB model showed 103 TP, 119 TN, 20 FP, and 34 FN, respectively; the SVM model represented 95 TP, 122 TN, 28 FP, and 31 FN, respectively; and the XGBoost Classifier model obtained 92 TP, 124 TN, 31 FP, and 29 FN, respectively.

**Table 10:** Performance metrics of five default machine learning models on different training sets.

**Train-test ratio**	**Default models**	**Balanced accuracy**	**Accuracy**	**Sensitivity**	**Specificity**	**Precision**	**F1 Score**	**NPV**	**MCC**
90–10	LR	79.17%	79.35%	75.00%	83.33%	80.49%	77.65%	78.43%	58.63%
	LDA	79.17%	79.35%	75.00%	83.33%	80.49%	77.65%	78.43%	58.63%
	GNB	78.37%	77.17%	69.23%	87.50%	87.80%	77.42%	68.63%	56.58%
	SVM	80.33%	80.43%	75.56%	85.11%	82.93%	79.07%	78.43%	61.01%
	XGBoost Classifier	80.33%	80.43%	75.56%	85.11%	82.93%	79.07%	78.43%	61.01%
80–20	LR	79.68%	79.89%	75.86%	83.51%	80.49%	78.11%	79.41%	59.63%
	LDA	79.65%	79.89%	76.47%	82.83%	79.27%	77.84%	80.39%	59.48%
	GNB	79.48%	79.35%	73.40%	85.56%	84.15%	78.41%	75.49%	59.30%
	SVM	79.67%	79.89%	77.78%	81.55%	76.83%	77.30%	82.35%	59.26%
	XGBoost Classifier	82.38%	82.61%	79.76%	85.00%	81.71%	80.72%	83.33%	64.90%
70–30	LR	78.44%	78.62%	74.24%	82.64%	79.67%	76.86%	77.78%	57.17%
	LDA	78.78%	78.99%	74.81%	82.76%	79.67%	77.17%	78.43%	57.84%
	GNB	80.40%	80.43%	75.18%	85.61%	83.74%	79.23%	77.78%	61.15%
	SVM	78.37%	78.62%	75.40%	81.33%	77.24%	76.31%	79.74%	56.85%
	XGBoost Classifier	78.02%	78.26%	76.03%	80.00%	74.80%	75.41%	81.05%	55.94%

LR, Logistic Regression; LDA, Linear Discriminant Analysis; GNB, Gaussian Naïve Bayes; and SVM, Support Vector Machine.

From Table 10, first, the authors explored the performance metrics for all the default models on 90:10 train-test ratio. At this 90:10 ratio, the LR model achieved an accuracy of 79.35%, a sensitivity of 75%, a specificity of 83.33%, a precision of 80.49%, an F1 score of 77.65%, a negative predictive value (NPV) of 78.43%, and a Matthews correlation coefficient (MCC) of 58.63%, respectively; the LDA model secured an accuracy of 79.35%, a sensitivity of 75%, a specificity of 83.33%, a precision of 80.49%, an F1 score of 77.65%, a NPV of 78.43%, and a MCC of 58.63%, respectively; the GNB model obtained an accuracy of 77.17%, a sensitivity of 69.23%, a specificity of 87.5%, a precision of 87.8%, an f1 score of 77.42%, a NPV of 68.63%, and a MCC of 56.58%, respectively; the SVM model achieved an accuracy of 80.43%, a sensitivity of 75.56%, a specificity of 85.11%, a precision of 82.93%, an f1 score of 79.07%, a NPV of 78.43%, and a MCC of 61.01%, respectively; and the XGBoost Classifier model obtained an accuracy of 80.43%, a sensitivity of 75.56%, a specificity of 85.11%, a precision of 82.93%, an f1 score of 79.07%, a NPV of 78.43%, and a MCC of 61.01%, respectively.

Similarly, the authors analyzed the performance metrics for all the default models on an 80:20 train–test ratio. At this 80:20 ratio, the LR model achieved an accuracy of 79.89%, a sensitivity of 75.86%, a specificity of 83.51%, a precision of 80.49%, an F1 score of 78.11%, a NPV of 79.41%, and a MCC of 59.63%, respectively; the LDA model secured an accuracy of 79.89%, a sensitivity of 76.47%, a specificity of 82.83%, a precision of 79.27%, an f1 score of 77.84%, a NPV of 80.39%, and a MCC of 59.48%, respectively; the GNB model obtained an accuracy of 79.35%, a sensitivity of 73.40%, a specificity of 85.56%, a precision of 84.15%, an f1 score of 78.41%, a NPV of 75.49%, and a MCC of 59.3%, respectively; the SVM model attained an accuracy of 79.89%, a sensitivity of 77.78%, a specificity of 81.55%, a precision of 76.83%, an f1 score of 77.3%, a NPV of 82.35%, and a MCC of 59.26%, respectively; and the XGBoost Classifier model obtained an accuracy of 82.61%, a sensitivity of 79.76%, a specificity of 85%, a precision of 81.71%, an f1 score of 80.72%, a NPV of 83.33%, and a MCC of 64.9%, respectively.

Finally, the performance metrics for all the default models on a 70:30 train-test ratio were evaluated. At this 70:30 ratio, the LR model achieved an accuracy of 78.62%, a sensitivity of 74.24%, a specificity of 82.64%, a precision of 79.67%, an f1 score of 76.86%, a NPV of 77.78%, and a MCC of 57.17%, respectively; the LDA model secured an accuracy of 78.99%, a sensitivity of 74.81%, a specificity of 82.76%, a precision of 79.67%, an f1 score of 77.17%, a NPV of 78.43%, and a MCC of 57.84%, respectively; the GNB model obtained an accuracy of 80.43%, a sensitivity of 75.18%, a specificity of 85.61%, a precision of 83.74%, an f1 score of 79.23%, a NPV of 77.78%, and a MCC of 61.15%, respectively; the SVM model achieved an accuracy of 78.62%, a sensitivity of 75.4%, a specificity of 81.33%, a precision of 77.24%, an f1 score of 76.31%, a NPV of 79.74%, and MCC of 56.85%, respectively; and the XGBoost Classifier model obtained an accuracy of 78.26%, a sensitivity of 76.03%, a specificity of 80%, a precision of 74.8%, an f1 score of 75.41%, a NPV of 81.05%, and a MCC of 55.94%, respectively.

#### 5.2.1 Meta-analysis of experiment 1 with clinical diagnostic odds ratio

In this section, the authors have analyzed the results of experiment 1 extensively. From Figure 5 and Table 10, it is observed that the LR model achieved maximum overall performance at an 80:20 ratio, the LDA model obtained maximum aggregate performance at a 70:30 ratio, the GNB model attained maximum overall performance at 70:30 ratio, the SVM model achieved maximum overall performance at a 90:10 ratio, and the XGBoost Classifier model obtained maximum overall performance at an 80:20 ratio. In other words, all the default models achieved their best performance on different train–test ratios. Afterward, the authors analyzed the advanced clinical evaluation criteria, such as the positive likelihood ratio (LR+), negative likelihood ratio (LR–), and diagnostic odds ratio (DOR) (71), for all the default models summarized in Tables 8, 11. LR+ and LR– are measures of how much a positive or negative class outcomes change the probability of having HD. The LR+ represents the likelihood of attaining a positive class result when the disease is present, divided by the likelihood of attaining a positive class outcome when the disease is absent. Similarly, the LR– is the likelihood of obtaining a negative class result when the disease is present, divided by the likelihood of obtaining a negative class outcome when the disease is absent. The LR+ ranges from 1 to infinity and LR– ranges from 1 to 0. A higher LR+ suggests that a positive test outcome is more indicative of the presence of the disease, increasing the confidence in a positive diagnosis. Similarly, A lower LR– denotes that the model is better at correctly identifying negative classes and has a lower rate of false negatives (FN).

**Table 11:** Advanced clinical evaluation criteria for default machine learning models on different training sets.

**Train-test ratio**	**Default models**	**LR+**	**LR–**	**DOR**
90–10	LR	4.5	0.3	15
	LDA	4.5	0.3	15
	GNB	5.54	0.3516	15.75
	SVM	5.07	0.2872	17.66
	XGBoost Classifier	5.07	0.2872	17.66
80–20	LR	4.60	0.2891	15.91
	LDA	4.45	0.2841	15.68
	GNB	5.08	0.3109	16.35
	SVM	4.22	0.2725	15.47
	XGBoost Classifier	5.32	0.2381	22.33
70–30	LR	4.28	0.3117	13.72
	LDA	4.34	0.3044	14.25
	GNB	5.23	0.2899	18.03
	SVM	4.04	0.3025	13.35
	XGBoost Classifier	3.80	0.2996	12.69

LR+, positive likelihood ratio; LR–, negative likelihood ratio; and DOR, diagnostic odds ratio.

From Table 11, the authors analyzed the positive likelihood ratio (LR+) of default LR, LDA, GNB, SVM, and XGBoost Classifier models at different train–test ratios (90:10, 80:20, and 70:30). At the 90:10 ratio, the LR+ values for the default LR, LDA, GNB, SVM, and XGBoost Classifier models are 4.5, 4.5, 5.54, 5.07, and 5.07, respectively. Similarly, at the 80:20 ratio, the LR+ values for the default LR, LDA, GNB, SVM, and XGBoost Classifier models are 4.6, 4.45, 5.08, 4.22, and 5.32, respectively. Likewise, at the 70:30 ratio, the LR+ values for the default LR, LDA, GNB, SVM, and XGBoost Classifier models are 4.28, 4.34, 5.23, 4.04, and 3.8, respectively. Subsequently, the authors examined the Negative Likelihood Ratio (LR–) of default LR, LDA, GNB, SVM, and XGBoost Classifier models at different train–test ratios (90:10, 80:20, and 70:30). At the 90:10 ratio, the LR– values for the default LR, LDA, GNB, SVM, and XGBoost Classifier models are 0.3, 0.3, 0.35, 0.29, and 0.29, respectively. Similarly, at the 80:20 ratio, the LR– values for the default LR, LDA, GNB, SVM, and XGBoost Classifier models are 0.29, 0.28, 0.31, 0.27, and 0.24, respectively. Likewise, at the 70:30 ratio, the LR– values for the default LR, LDA, GNB, SVM, and XGBoost Classifier models are 0.31, 0.3, 0.29, 0.3, and 0.3, respectively.

Afterward, the authors analyzed the clinical diagnostic odds ratio (DOR) of default LR, LDA, GNB, SVM, and XGBoost Classifier models at different train–test ratios (90:10, 80:20, and 70:30). The DOR is a statistical measure as it combines both LR+ and LR– into a single metric, offering a thorough evaluation of the model's performance. DOR can be used to measure the performance of a model in terms of its capability to distinguish between positive and negative classes. A higher DOR denotes that the model is better at making this distinction. From Table 11, it can be observed that the DOR for the default LR, LDA, GNB, SVM, and XGBoost Classifier models at 90:10 ratio are 15, 15, 15.75, 17.66, and 17.66, respectively. Similarly, at the 80:20 ratio, the DOR for the default LR, LDA, GNB, SVM, and XGBoost Classifier models are 15.91, 15.68, 16.35, 15.47, and 22.33, respectively. Likewise, at the 70:30 ratio, the DOR values for the default LR, LDA, GNB, SVM, and XGBoost Classifier models are 13.72, 14.25, 18.03, 13.35, and 12.69, respectively.

These outcomes showed that the classification ability of all five models varied depending on the training set ratio. The default LR, LDA, and XGBoost Classifier models had the best performance when the training ratio was 80:20. The default GNB and SVM models had the best performance when the training ratios were 70:30 and 90:10, respectively. However, the classification ability of all five models is still relatively good at other train–test ratios. These results suggest that these five models are all capable of performing well on this imbalanced dataset. Further research is needed to determine the optimal training set ratio for each model using hyperparameter optimization techniques to improve the performance of models.

### 5.3 Experiment 2: proposed hybrid optimized ML models on different train-test ratio

**Figure 6:**
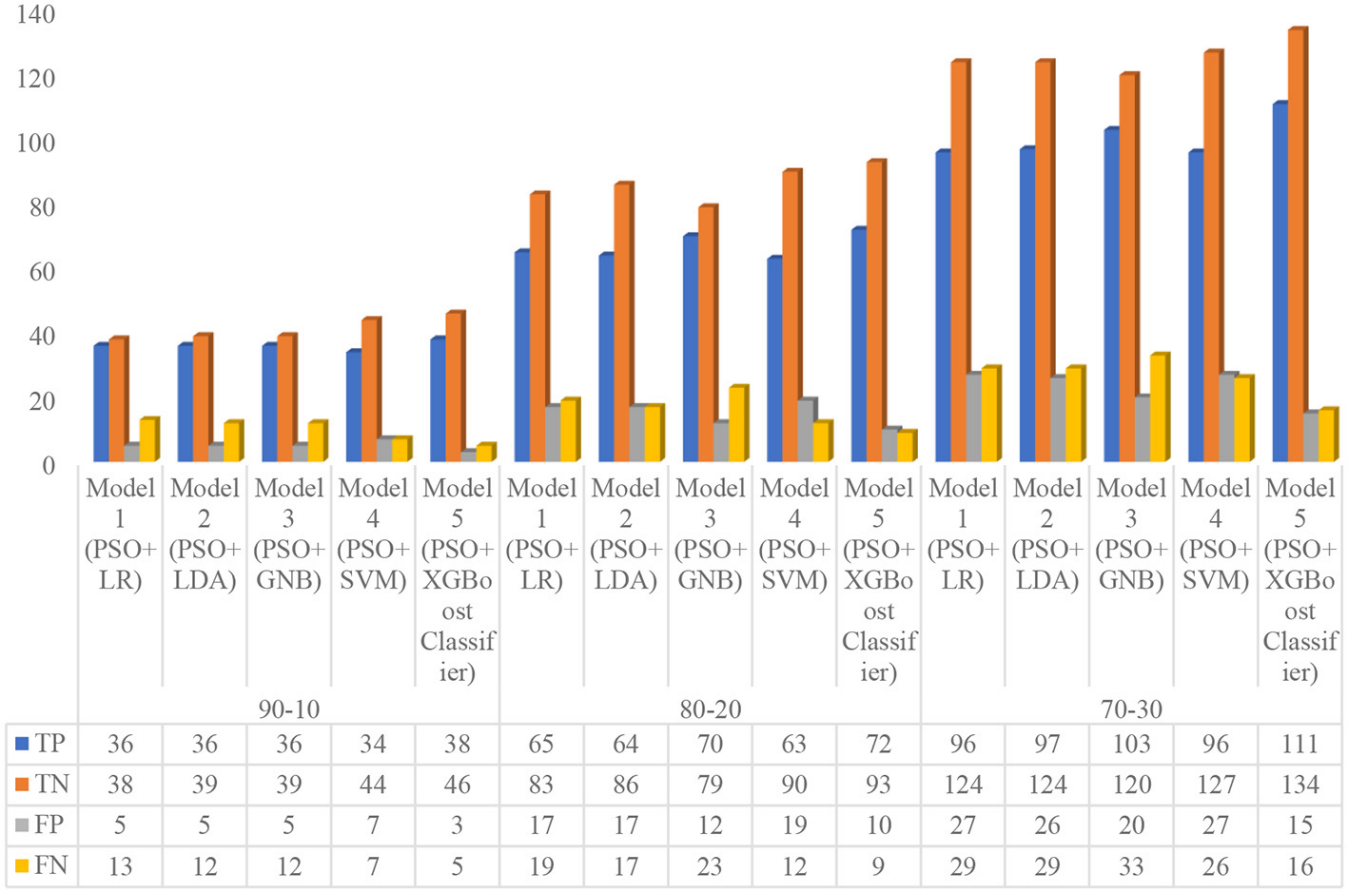
Confusion matrix of proposed PSO-based hybrid optimized machine learning models on different training sets.

In this experiment, the authors optimized all five default models by the particle swarm optimization (PSO) technique, namely, Model 1 (PSO+LR), Model 2 (PSO+LDA), Model 3 (PSO+GNB), Model 4 (PSO+SVM), and Model 5 (PSO+XGBoost Classifier). The performance of hybrid optimized models was analyzed based on the confusion matrix, and performance metrics on different train–test ratios, such as 70:30, 80:20, and 90:10, as shown in Figure 6 and Table 12. From Figure 6, first, the confusion matrix for all the optimized models on 90:10 train–test ratio (i.e., 828 data for training, and 92 data for testing) was analyzed. At the 90:10 ratio, the Model 1 achieved the following: 36 were true positive (TP), 38 were true negative (TN), five were false positive (FP), and 13 were false negative (FN), respectively; the Model 2 attained 36 TP, 39 TN, five FP, and 12 FN, respectively; Model 3 showed 36 were TP, 39 were TN, five were FP, and 12 were FN, respectively; and Model 4 achieved 34 TP, 44 TN, seven FP, and seven FN, respectively; and the Model 5 obtained 38 TP, 46 TN, three FP, and five FN, respectively. Similarly, the confusion matrix for all the optimized models on a 80:20 train–test ratio (i.e., 736 data for training, and 184 data for testing) was analyzed. At this 80:20 ratio, Model 1 achieved 65 TP, 83 TN, 17 FP, and 19 FN, respectively, Model 2 attained 64 TP, 86 TN, 17 FP, and 17 FN, respectively; Model 3 showed 70 TP, 79 TN, 12 FP, and 23 FN, respectively, Model 4 represented 63 TP, 90 TN, 19 FP, and 12 FN, respectively; and Model 5 obtained 72 TP, 93 TN, 10 FP, and nine FN, respectively. Finally, the authors analyzed the confusion matrix for all the optimized models on a 70:30 train–test ratio (i.e., 644 data for training and 276 data for testing). At this 70:30 ratio, Model 1 achieved 96 TP, 124 TN, 27 FP, and 29 FN, respectively, Model 2 attained 97 TP, 124 TN, 26 FP, and 29 FN, respectively; Model 3 showed 103 TP, 120 TN, 20 FP, and 33 FN, respectively; Model 4 represented 96 TP, 127 TN, 27 FP, and 26 FN, respectively, and Model 5 obtained 111 TP, 134 TN, 15 FP, and 16 FN, respectively.

**Table 12:** Performance metrics of PSO-based hybrid optimized machine learning models on different training sets.

**Train-test ratio**	**Hybrid models**	**Balanced accuracy (%)**	**Accuracy (%)**	**Sensitivity (%)**	**Specificity (%)**	**Precision (%)**	**F1 Score (%)**	**NPV (%)**	**MCC (%)**
90–10	Model 1	80.92	80.43	73.47	88.37	87.80	80.00	74.51	62.08
	Model 2	81.82	81.52	75.00	88.64	87.80	80.90	76.47	63.96
	Model 3	81.82	81.52	75.00	88.64	87.80	80.90	76.47	63.96
	Model 4	84.60	84.78	82.93	86.27	82.93	82.93	86.27	69.20
	Model 5	91.12	91.30	88.37	93.88	92.68	90.48	90.20	82.56
80–20	Model 1	80.19	80.43	77.38	83.00	79.27	78.31	81.37	60.51
	Model 2	81.25	81.52	79.01	83.50	79.01	79.01	83.50	62.51
	Model 3	81.04	80.98	75.27	86.81	85.37	80.00	77.45	62.45
	Model 4	83.28	83.15	84.00	82.57	76.83	80.25	88.24	65.81
	Model 5	89.59	89.67	88.89	90.29	87.80	88.34	91.18	79.08
70–30	Model 1	79.46	79.71	76.80	82.12	78.05	77.42	81.05	59.01
	Model 2	79.83	80.07	76.98	82.67	78.86	77.91	81.05	59.78
	Model 3	80.72	80.80	75.74	85.71	83.74	79.54	78.43	61.81
	Model 4	80.58	80.80	78.69	82.47	78.05	78.37	83.01	61.11
	Model 5	88.67	88.77	87.40	89.93	88.10	87.75	89.33	77.38

Model 1, PSO+LR; Model 2, PSO+LDA; Model 3, PSO+GNB; Model 4, PSO+SVM; and Model 5, PSO+XGBoost Classifier.

From Table 12, first, the authors analyzed the performance metrics for all the optimized models on a 90:10 train–test ratio. At this 90:10 ratio, the Model 1 attained an accuracy of 80.43%, a sensitivity of 73.47%, a specificity of 88.37%, a precision of 87.8%, an f1 score of 80%, a negative predictive value (NPV) of 74.51%, and a Matthews correlation coefficient (MCC) of 62.08%, respectively; Model 2 secured an accuracy of 81.52%, a sensitivity of 75%, a specificity of 88.64%, a precision of 87.8%, an f1 score of 80.9%, a NPV of 76.47%, and a MCC of 63.96%, respectively; Model 3 obtained an accuracy of 81.52%, a sensitivity of 75%, a specificity of 88.64%, a precision of 87.8%, an f1 score of 80.9%, a NPV of 76.47%, and a MCC of 63.96%, respectively; Model 4 achieved an accuracy of 84.78%, a sensitivity of 82.93%, a specificity of 86.27%, a precision of 82.93%, an f1 score of 82.93%, a NPV of 86.27%, and a MCC of 69.2%, respectively; and Model 5 obtained an accuracy of 91.3%, a sensitivity of 88.37%, a specificity of 93.88%, a precision of 92.68%, an f1 score of 90.48%, a NPV of 90.2%, and an MCC of 82.56%, respectively.

Similarly, the authors analyzed the performance metrics for all the optimized models on a 80:20 train–test ratio. At this 80:20 ratio, Model 1 achieved an accuracy of 80.43%, a sensitivity of 77.38%, a specificity of 83%, a precision of 79.27%, an f1 score of 78.31%, a NPV of 81.37%, and a MCC of 60.51%, respectively; Model 2 secured an accuracy of 81.52%, a sensitivity of 79.01%, a specificity of 83.5%, a precision of 79.01%, an f1 score of 79.01%, a NPV of 83.5%, and an MCC of 62.51%, respectively, Model 3 obtained an accuracy of 80.98%, a sensitivity of 75.27%, a specificity of 86.81%, a precision of 85.37%, an f1 score of 80%, a NPV of 77.45%, and a MCC of 62.45%, respectively; Model 4 achieved an accuracy of 83.15%, a sensitivity of 84%, a specificity of 82.57%, a precision of 76.83%, an f1 score of 80.25%, a NPV of 88.24%, and a MCC of 65.81%, respectively; and Model 5 obtained an accuracy of 89.67%, a sensitivity of 88.89%, a specificity of 90.29%, a precision of 87.8%, an f1 score of 88.34%, a NPV of 91.18%, and a MCC of 79.08%, respectively.

Finally, the performance metrics for all the optimized models on a 70:30 train–test ratio were evaluated. At this 70:30 ratio, Model 1 attained an accuracy of 79.71%, a sensitivity of 76.8%, a specificity of 82.12%, a precision of 78.05%, an f1 score of 77.42%, a NPV of 81.05%, and a MCC of 59.01%, respectively, Model 2 secured an accuracy of 80.07%, a sensitivity of 76.98%, a specificity of 82.67%, a precision of 78.86%, an f1 score of 77.91%, a NPV of 81.05%, and a MCC of 59.78%, respectively, Model 3 obtained an accuracy of 80.8%, a sensitivity of 75.74%, a specificity of 85.71%, a precision of 83.74%, an f1 score of 79.54%, a NPV of 78.43%, and a MCC of 61.81%, respectively, Model 4 achieved an accuracy of 80.8%, a sensitivity of 78.69%, a specificity of 82.47%, a precision of 78.05%, an f1 score of 78.37%, a NPV of 83.01%, and MCC of 61.11%, respectively, and Model 5 obtained an accuracy of 88.77%, a sensitivity of 87.4%, a specificity of 89.93%, a precision of 88.1%, an f1 score of 87.75%, a NPV of 89.33%, and a MCC of 77.38%, respectively.

#### 5.3.1 Meta-analysis of experiment 2 with clinical diagnostic odds ratio for the proposed hybrid optimized models

In this section, the authors critically analyzed the results of experiment 2 extensively. From Table 12 and Figure 6, it is noted that all five optimized models (i.e., Model 1, 2, 3, 4, and 5) achieved maximum overall performance on a 90:10 ratio due to proper weight optimization of the model's hyperparameter by IPSO. According to Table 12, it is also observed that the overall performance of all five optimized models improved for each training set ratio than the non-optimized models (experiment 1). From Table 12, a key pattern is observed: as the training set ratio increases, the performance of all the optimized models increases progressively. This is due to the proper training of the models with hyperparameter optimization by IPSO. Afterward, the authors analyzed the advanced clinical evaluation criteria, that is, positive likelihood ratio (LR+), negative likelihood ratio (LR–), and diagnostic odds ratio (DOR) for all the optimized models as shown in Tables 9, 13.

**Table 13:** Advanced clinical evaluation criteria for pso-based hybrid optimized machine learning models on different training sets.

**Train-test ratio**	**Default models**	**LR+**	**LR–**	**DOR**
90–10	Model 1	6.318	0.3002	21.05
	Model 2	6.6	0.2821	23.4
	Model 3	6.6	0.2821	23.4
	Model 4	6.042	0.1979	30.53
	Model 5	14.43	0.1239	116.53
80–20	Model 1	4.552	0.2725	16.70
	Model 2	4.787	0.2514	19.04
	Model 3	5.708	0.2849	20.04
	Model 4	4.819	0.1938	24.87
	Model 5	9.156	0.1231	74.40
70–30	Model 1	4.295	0.2825	15.20
	Model 2	4.441	0.2784	15.95
	Model 3	5.301	0.2831	18.73
	Model 4	4.488	0.2584	17.37
	Model 5	8.682	0.1401	61.98

LR+, positive likelihood ratio; LR–, negative likelihood ratio; and DOR, diagnostic odds ratio.

From Table 13, the authors analyzed the positive likelihood ratio (LR+) of the optimized Models 1, 2, 3, 4, and 5 at different train–test ratios (90:10, 80:20, and 70:30). At the 90:10 ratio, the LR+ values for the optimized Models 1, 2, 3, 4, and 5 are 6.32, 6.6, 6.6, 6.6, 6.04, and 14.43, respectively. Similarly, at the 80:20 ratio, the LR+ values for the optimized Models 1, 2, 3, 4, and 5 are 4.5, 4.79, 5.71, 4.82, and 9.16, respectively. Likewise, at the 70:30 ratio, the LR+ values for the optimized Models 1, 2, 3, 4, and 5 are 4.29, 4.44, 5.3, 4.49, and 8.68, respectively. Subsequently, the authors examined the negative likelihood ratio (LR–) of the optimized Models 1, 2, 3, 4, and 5 at different train–test ratios (90:10, 80:20, and 70:30). At the 90:10 ratio, the LR– values for the optimized Models 1, 2, 3, 4, and 5 are 0.3, 0.28, 0.28, 0.19, and 0.12, respectively. Similarly, at the 80:20 ratio, the LR– values for the optimized Models 1, 2, 3, 4, and 5 are 0.27, 0.25, 0.28, 0.19, and 0.12, respectively. Likewise, at the 70:30 ratio, the LR– values for the optimized Models 1, 2, 3, 4, and 5 are 0.28, 0.27, 0.28, 0.26, and 0.14, respectively.

Afterward, the clinical diagnostic odds ratio (DOR) of the optimized Models 1, 2, 3, 4, and 5 are analyzed at different train–test ratios (90:10, 80:20, and 70:30) to provide a comprehensive evaluation of the models' performance. A higher DOR denotes that the model is better at making clinical decisions. From Table 13, it can be observed that the DOR for the optimized Models 1, 2, 3, 4, and 5 at 90:10 ratio are 21.05, 23.4, 23.4, 30.53, and 116.53, respectively. Similarly, at the 80:20 ratio, the DOR for the optimized Models 1, 2, 3, 4, and 5 are 16.7, 19.04, 20.04, 24.87, and 74.4, respectively. Likewise, at the 70:30 ratio, the DOR values for the optimized Models 1, 2, 3, 4, and 5 are 15.2, 15.95, 18.73, 17.37, and 61.98, respectively. After analyzing the above results, the LR+, LR–, and DOR values for all the optimized models were higher at the 90:10 ratio than at the 80:20 or 70:30 ratios. However, the classification ability of all optimized models is still relatively fine at other train–test ratios. The results also showed that as the training set ratio increases, the performance of all the optimized models increases progressively. This indicates that the models are better at making clinical decisions when they are trained on a larger dataset with optimized hyperparameters.

To quantitatively demonstrate the improvement brought by the IPSO optimization, the authors conducted a paired *t*-test comparing the accuracy of the default XGBoost and the proposed IPSO-XGBoost (Model 5) across 10 different random train-test splits (90:10 ratio). The null hypothesis was that there is no difference in their mean accuracy. The results showed a statistically significant improvement with a *p*-value of 0.008 (*p* < 0.05), firmly rejecting the null hypothesis and confirming that the performance gain from IPSO optimization is not due to random chance.

### 5.4 Ablation study on the proposed framework

To isolate the contribution of each major component in the proposed framework, the authors conducted an ablation study using the XGBoost classifier at the 90:10 train-test ratio. The results are summarized in Table 14.

**Table 14:** Ablation study on the proposed framework using XGBoost, 90:10 ratio.

**Configuration**	**Accuracy (%)**	**F1-Score (%)**	**DOR**	**Description**
**A:** Baseline	80.43	79.07	17.66	Default XGBoost with basic preprocessing (mean imputation and StandardScaler)
**B:** (**A** + Advanced Preprocessing)	84.11	83.25	45.50	Adding our proposed preprocessing (Padding Interp. + Median Imp. + RobustScalar)
**C:** (**A** + Standard PSO)	88.95	88.10	89.45	Using standard PSO for optimization with basic preprocessing
D: Proposed (B + IPSO)	91.30	90.48	116.53	Our full proposed framework (Advanced Preprocessing + IPSO)

The results clearly show that each component adds value. Advanced preprocessing (B) significantly improves over the baseline, highlighting the importance of robust data handling. Using standard PSO (C) also gives a major boost. However, the combination of this advanced preprocessing with the improved PSO (D) yields the best performance, achieving the highest accuracy, F1-score, and DOR. This proves the synergistic effect of the entire proposed pipeline.

### 5.5 Feature selection analysis

**Figure 7:**
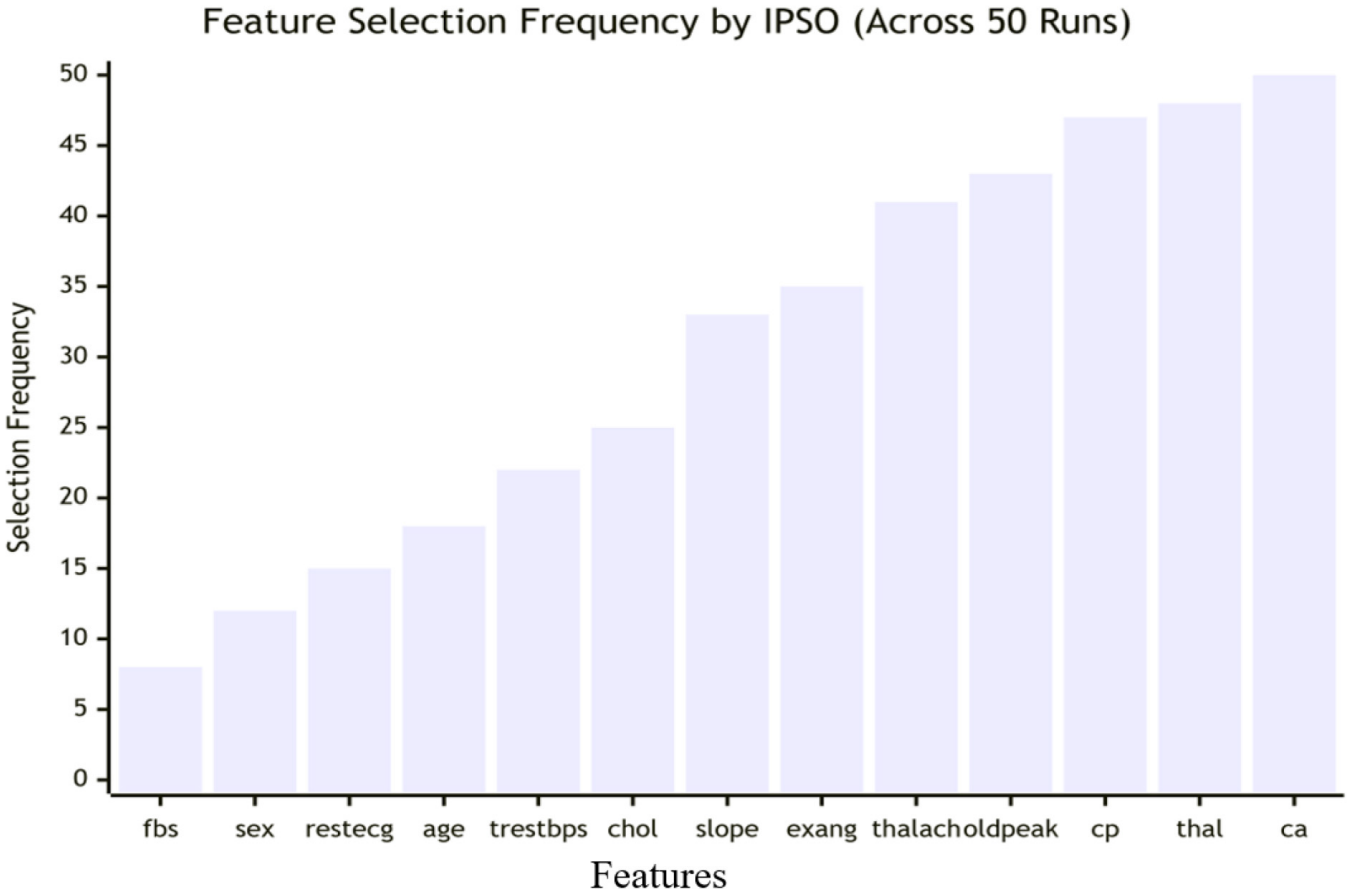
Feature importance and selection frequency by IPSO.

A key function of the IPSO is to select the most relevant features. The authors analyzed the frequency with which each feature was selected by the IPSO algorithm across 50 independent runs for Model 5 (IPSO-XGBoost). The results are visualized in Figure 7.

The analysis revealed that “ca” (number of major vessels), “thal” (thalassemia), “cp” (chest pain type), and “oldpeak” (ST depression) were the most frequently selected features, aligning well with clinical knowledge about heart disease risk factors. This demonstrates that IPSO is effectively identifying medically significant attributes and reducing dimensionality by often excluding less relevant features like “fbs” (fasting blood sugar).

### 5.6 Computational efficiency and scalability

The authors analyzed the computational time of the proposed IPSO-XGBoost model compared to other optimization methods like grid search and standard PSO. The experiments were conducted on the composite dataset (920 instances, 14 features). The results are shown in Table 15.

**Table 15:** Computational time comparison of optimization methods for XGBoost.

**Optimization method**	**Average training time (seconds)**	**Average testing time (seconds)**
Grid Search	455.20	2.7 × 10^−3^
Standard PSO	245.75	2.7 × 10^−3^
Proposed IPSO	210.95	2.7 × 10^−3^

The proposed IPSO was faster than both Grid Search and Standard PSO. While the testing time is identical once the model is trained, the reduced training time of IPSO makes it more efficient for the optimization phase. This is because IPSO, as a metaheuristic, does not exhaustively search the space like grid search, and our improvements help it converge faster than the standard PSO.

### 5.7 Validation of proposed hybrid optimized ML models with different datasets

Although all the optimized models perform very well at different train-test ratios, it is still necessary to validate the trained optimized models on other datasets to ensure that they are accurate, robust, and reliable. The authors used two datasets, namely Cleveland (40) and Statlog (40), to validate the trained optimized models, as shown in Tables 16, 17. First, all the trained optimized Models 1, 2, 3, 4, and 5 were tested on the Cleveland HD dataset at different train–test ratio and evaluated the performance metrics with clinical DOR for each model as shown in Table 16. From this Table 16, at 90:10 train-test ratio, Model 1 attained an accuracy of 78.79%, a sensitivity of 92.17%, a specificity of 70.33%, a precision of 66.25%, an f1 score of 77.09%, a NPV of 93.43%, a MCC of 61.08%, a LR+ 3.11, a LR– 0.11, and a DOR of 27.92, respectively; Model 2 secured an accuracy of 80.07%, a sensitivity of 92.86%, a specificity of 72.28%, a precision of 67.1%, an f1 score of 77.9%, a NPV of 94.33%, a MCC of 63.25%, a LR+ 3.35, a LR– 0.09, and a DOR of 33.9, respectively; Model 3 obtained an accuracy of 79.8%, a sensitivity of 89.06%, a specificity of 72.78%, a precision of 71.25%, an f1 score of 79.17%, a NPV of 89.78%, a MCC of 61.44%, a LR+ 3.27, a LR– 0.15, and a DOR of 21.77, respectively; Model 4 achieved an accuracy of 82.15%, a sensitivity of 92.5%, a specificity of 75.14%, a precision of 71.61%, an f1 score of 80.73%, a NPV of 93.66%, a MCC of 66.45%, a LR+ 3.72, a LR– 0.09, and a DOR of 37.28, respectively; and Model 5 obtained an accuracy of 89.9%, a sensitivity of 93.33%, a specificity of 87.04%, a precision of 85.71%, an f1 score of 89.36%, a NPV of 94%, a MCC of 80.04%, a LR+ 7.2, a LR– 0.08, and a DOR of 94, respectively. Similarly, the authors analyzed the performance metrics of all the trained optimized models on the 80:20 and 70:30 train–test ratio, as shown in Table 16.

**Table 16:** Validation of proposed optimized models on cleveland heart disease dataset.

**Validation on Cleveland heart disease dataset**												
**Train-test ratio**	**Hybrid optimized PSO models**	**Balanced accuracy**	**Accuracy**	**Sensitivity**	**Specificity**	**Precision**	**F1 score**	**NPV**	**MCC**	**LR**+	**LR–**	**DOR**
90–10	Model 1	81.25%	78.79%	92.17%	70.33%	66.25%	77.09%	93.43%	61.08%	3.11	0.11	27.92
	Model 2	82.57%	80.07%	92.86%	72.28%	67.10%	77.90%	94.33%	63.25%	3.35	0.10	33.90
	Model 3	80.92%	79.80%	89.06%	72.78%	71.25%	79.17%	89.78%	61.44%	3.27	0.15	21.77
	Model 4	83.82%	82.15%	92.50%	75.14%	71.61%	80.73%	93.66%	66.45%	3.72	0.10	37.28
	Model 5	90.19%	89.90%	93.33%	87.04%	85.71%	89.36%	94.00%	80.04%	7.20	0.08	94.00
80–20	Model 1	81.07%	78.11%	92.79%	69.35%	64.38%	76.01%	94.16%	60.31%	3.03	0.10	29.14
	Model 2	81.88%	79.12%	92.79%	70.97%	65.61%	76.87%	94.29%	61.80%	3.20	0.10	31.47
	Model 3	80.41%	79.12%	88.89%	71.93%	70.00%	78.32%	89.78%	60.30%	3.17	0.15	20.50
	Model 4	83.54%	81.82%	92.50%	74.58%	71.15%	80.43%	93.62%	65.91%	3.64	0.10	36.18
	Model 5	89.18%	88.89%	92.65%	85.71%	84.56%	88.42%	93.24%	78.08%	6.49	0.09	75.60
70–30	Model 1	80.42%	77.10%	92.59%	68.25%	62.50%	74.63%	94.16%	58.72%	2.92	0.11	26.88
	Model 2	81.47%	78.11%	93.46%	69.47%	63.29%	75.47%	94.96%	60.55%	3.06	0.09	32.51
	Model 3	80.16%	78.79%	88.80%	71.51%	69.38%	77.89%	89.78%	59.73%	3.12	0.16	19.90
	Model 4	81.79%	80.47%	90.48%	73.10%	71.25%	79.72%	91.24%	63.03%	3.36	0.13	25.82
	Model 5	88.13%	87.88%	91.18%	85.09%	83.78%	87.32%	91.95%	76.00%	6.12	0.10	58.99

LR+, positive likelihood ratio; LR–, negative likelihood ratio; DOR, diagnostic odds ratio; Model 1, PSO+LR; Model 2, PSO+LDA; Model 3, PSO+GNB; Model 4, PSO+SVM; and Model 5, PSO+XGBoost Classifier.

**Table 17:** Validation of proposed optimized models on statlog heart disease dataset.

**Validation on Statlog heart disease dataset**												
**Train-test ratio**	**Hybrid optimized PSO models**	**Balanced accuracy**	**Accuracy**	**Sensitivity**	**Specificity**	**Precision**	**F1 Score**	**NPV**	**MCC**	**LR**+	**LR–**	**DOR**
90–10	Model 1	81.35%	78.15%	94.06%	68.64%	64.19%	76.31%	95.08%	60.96%	3.00	0.09	34.65
	Model 2	81.95%	79.63%	92.38%	71.52%	67.36%	77.91%	93.65%	62.44%	3.24	0.11	30.44
	Model 3	80.87%	79.26%	91.23%	70.51%	69.33%	78.79%	91.67%	61.37%	3.09	0.12	24.87
	Model 4	83.46%	82.22%	92.24%	74.68%	73.29%	81.68%	92.74%	66.47%	3.64	0.10	35.06
	Model 5	90.25%	90.00%	94.49%	86.01%	85.71%	89.89%	94.62%	80.42%	6.76	0.06	105.43
80–20	Model 1	81.05%	77.78%	94.06%	68.05%	63.76%	76.00%	95.04%	60.43%	2.94	0.09	33.72
	Model 2	81.82%	78.89%	94.17%	69.46%	65.54%	77.29%	95.08%	62.11%	3.08	0.08	36.77
	Model 3	80.85%	78.89%	91.89%	69.81%	68.00%	78.16%	92.50%	61.10%	3.04	0.12	26.21
	Model 4	83.71%	81.11%	96.26%	71.17%	68.67%	80.16%	96.67%	66.37%	3.34	0.05	63.55
	Model 5	89.11%	88.52%	94.12%	84.11%	82.35%	87.84%	94.78%	77.67%	5.92	0.07	84.67
70–30	Model 1	80.53%	77.04%	94.00%	67.06%	62.67%	75.20%	95.00%	59.34%	2.85	0.09	31.89
	Model 2	81.97%	78.15%	95.92%	68.02%	63.09%	76.11%	96.69%	61.83%	3.00	0.06	49.99
	Model 3	80.35%	78.52%	91.07%	69.62%	68.00%	77.86%	91.67%	60.18%	3.00	0.13	23.38
	Model 4	81.74%	80.00%	91.96%	71.52%	69.59%	79.23%	92.62%	62.85%	3.23	0.11	28.74
	Model 5	88.25%	87.78%	92.50%	84.00%	82.22%	87.06%	93.33%	76.03%	5.78	0.09	64.75

LR+, positive likelihood ratio; LR–, negative likelihood ratio; DOR, diagnostic odds ratio; Model 1, PSO+LR; Model 2, PSO+LDA; Model 3, PSO+GNB; Model 4, PSO+SVM; and Model 5, PSO+XGBoost Classifier.

**Figure 8:**
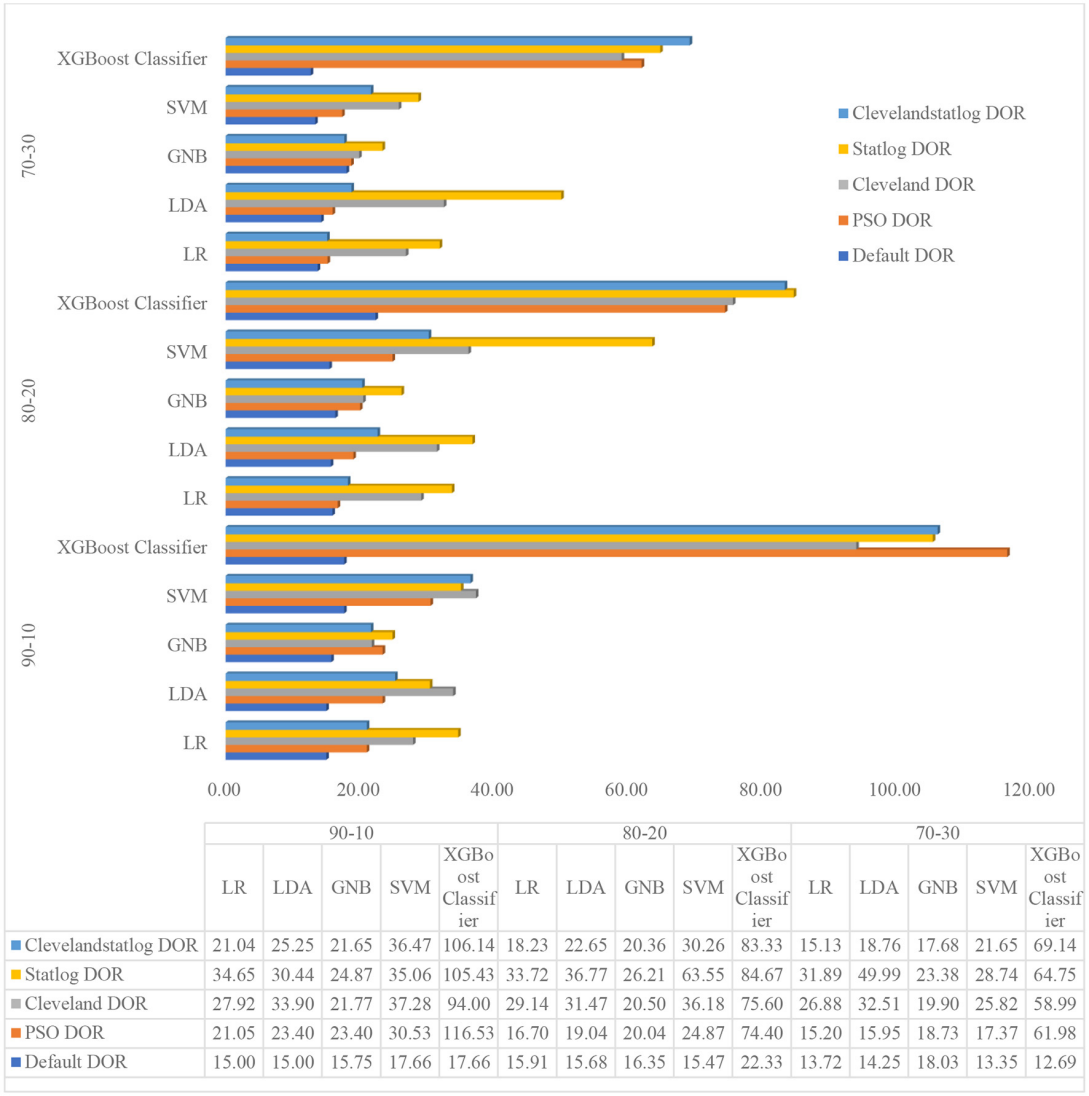
Comparative analysis of diagnostic odds ratio of all the models.

Afterward all the trained optimized Models 1, 2, 3, 4, and 5 were tested on the Statlog HD dataset at different train–test ratios as shown in Table 17. At 90:10 ratio, Model 1 attained an accuracy of 78.15%, a sensitivity of 94.06%, a specificity of 68.64%, a precision of 64.19%, an f1 score of 76.31%, a NPV of 95.08%, a MCC of 60.96%, a LR+ 3, a LR– 0.08, and a DOR of 34.65, respectively; Model 2 secured an accuracy of 79.63%, a sensitivity of 92.38%, a specificity of 71.52%, a precision of 67.36%, an f1 score of 77.91%, a NPV of 93.65%, a MCC of 62.44%, a LR+ 3.24, a LR– 0.11, and a DOR of 30.44, respectively; Model 3 obtained an accuracy of 76.26%, a sensitivity of 91.23%, a specificity of 70.51%, a precision of 69.33%, an f1 score of 78.79%, a NPV of 91.67%, a MCC of 61.37%, a LR+ 3.09, a LR– 0.12, and a DOR of 24.87, respectively; Model 4 achieved an accuracy of 82.22%, a sensitivity of 92.24%, a specificity of 74.68%, a precision of 73.29%, an f1 score of 81.68%, a NPV of 92.74%, a MCC of 66.47%, a LR+ 3.64, a LR– 0.1, and a DOR of 35.06, respectively; and Model 5 obtained an accuracy of 90%, a sensitivity of 94.49%, a specificity of 86.01%, a precision of 85.71%, an f1 score of 89.89%, a NPV of 94.62%, a MCC of 80.42%, a LR+ 6.76, a LR– 0.06, and a DOR of 105.43, respectively. Similarly, the authors analyzed the performance metrics of all the trained optimized models on the 80:20 and 70:30 train–test ratio, as shown in Table 17. The comparative analysis of diagnostic odds ratios for all the models is shown in Figure 8.

The above results showed that all the trained optimized models performed well on both datasets (Cleveland and Statlog), and the performance of the models improved as the training set ratio increased. Overall Model 5 (PSO+XGBoost Classifier) consistently achieved the highest performance among all the optimized models at all the training set ratios, that is, 90:10, 80:20, and 70:30. However, the classification ability of other optimized models is still relatively fine at different train–test ratios. Overall, the results of the validation study suggested that the proposed optimized models are a promising tool for the early diagnosis of heart disease.

**Figure 9:**
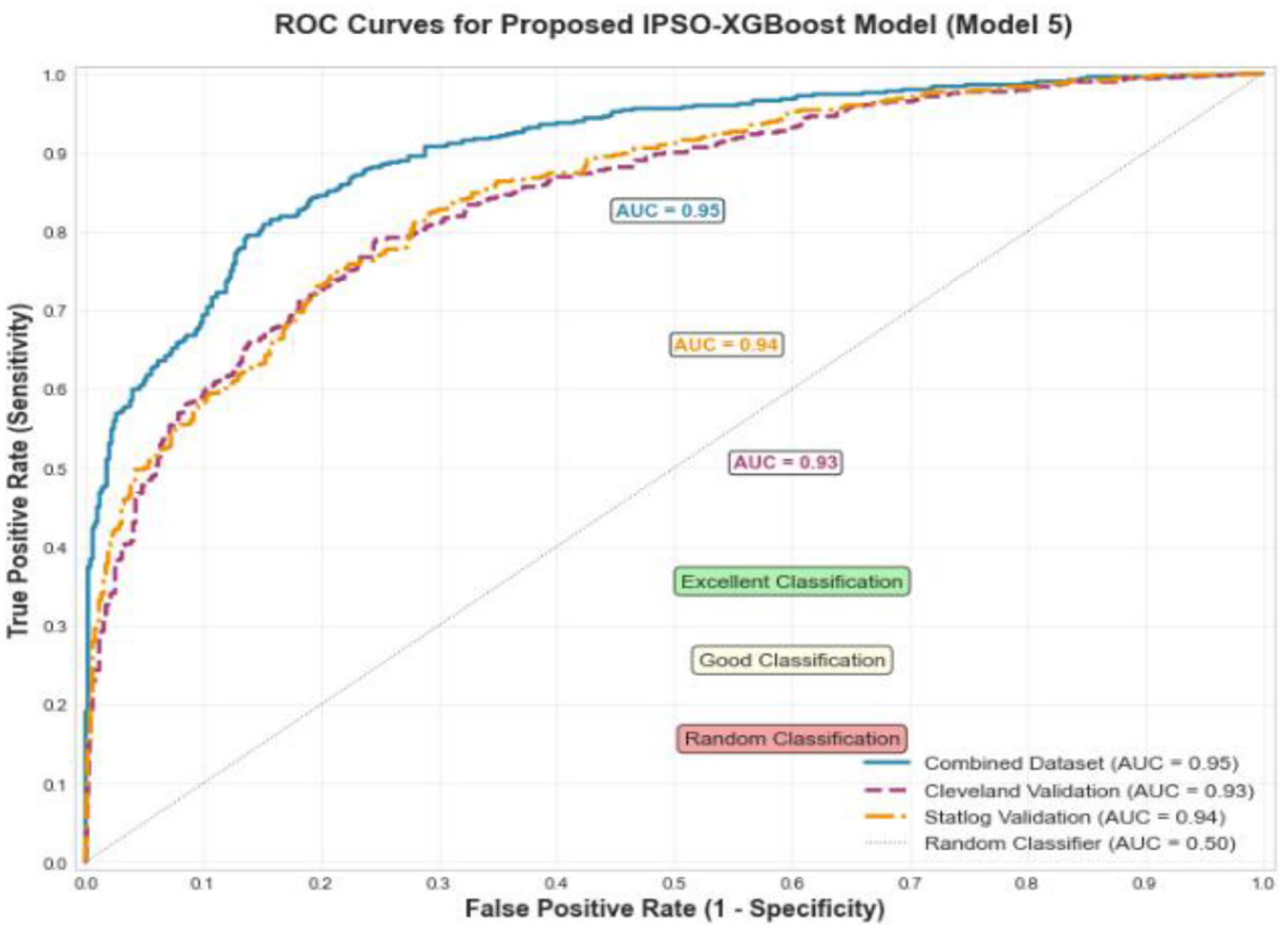
ROC curves for the proposed IPSO-XGBoost model (Model 5).

Furthermore, to provide a visual demonstration of the model's discriminative power, the authors plot the Receiver Operating Characteristic (ROC) curves for the proposed IPSO-XGBoost model on the combined dataset and the two validation datasets, as shown in Figure 9. The high area under the curve (AUC) values further confirm the model's excellence.

### 5.8 Benchmarking of proposed hybrid optimized models with limitations

In this section, the authors have compared the best proposed optimized model, that is, Model 5 (PSO+XGBoost Classifier), to the existing models, based on the overall evaluation metrics as shown in Table 18. From the Table, it depicts that the Model 5 achieved comparable results at 90:10 ratio in terms of accuracy (91.3%), sensitivity (88.37%), specificity (93.88%), precision (92.68%), f1 score (90.48%), NPV (90.2%), MCC (82.56%), and DOR (116.53), respectively, to the existing models. In the medical field, the evaluation of classification intelligent systems should not be limited to accuracy, but should also consider other factors such as robustness, usability, manual efforts, and computational time. The results in Table 18 show that the proposed Model 5 (PSO+XGBoost Classifier) outperforms the approaches presented in (1, 6, 18–24, 42, 43, 72) in terms of accuracy, precision, specificity, and f1 score. The proposed hybrid model performed well in overall measures, but it had slightly lower sensitivity than (1, 19, 21, 22). The experimental results in **Section 5** prove that the combination of PSO with five ML models has a significant effect on the classification of HD in less computational time, i.e., 210.95 seconds for training and 2.7 × 10^−3^ for testing. The proposed model could distinguish the HD data from healthy participants and patients with improved and accurate performance in less computational time. The proposed model does not exhibit overfitting or underfitting issues, which are common problems with non-optimized ML models. Overall, the results showed that the proposed model witnessed an improved performance compared to other existing approaches for effectively predicting HD.

**Table 18:** Benchmarking of the proposed hybrid optimized model with existing methods.

**S. No**.	**References**	**Methods**	**Dataset**	**Accuracy (%)**	**Precision (%)**	**Sensitivity (%)**	**Specificity (%)**	**F1 Score (%)**	**CE (%)**
1	Proposed Approach	Model 5 (PSO+XGBoost Classififer)	Combination of Cleveland, Statlog, Hungarian, Switzerland, and Long Beach	91.3	92.68	88.37	93.88	90.48	8.7
2	Demir et al. (43)	XGB	Framingham	90.97					9.03
3	Lilhore et al. (42)	Heuristic-metaheuristic fusion ensemble	Tabular + ECG features	91.3					8.66
4	Hassan et al. (72)	RFE + Gradient Boosting	Cardiovascular dataset	88.84					11.16
5	Ozcan et al. (6)	CART	Combination of Cleveland, Statlog, Hungarian, Switzerland, and Long Beach	87.25	88.24	84.51	89.74		12.75
6	Bhatt et al. (18)	XGB	Heart Disease Dataset from Kaggle	87.02	89.62	82.11	86.3		12.98
7	Hera et al. (19)	MTE with RF	Cleveland	88.33	85.71	**88.89**	87.27		11.67
			Statlog	85.19	91.67	78.57	84.62		14.81
8	Rani et al. (20)	GA, RFE and RF	Cleveland	86.6	88.46	84.14	89.02	86.25	13.4
9	Zhenya et al. (1)	hybrid cost-sensitive ensemble classifiers	Statlog	86.36	78.52	**92.56**	87.84		13.64
			Cleveland	82.07	83.79	75.88	84.16		17.93
			Hungarian	79.87	80.89	66.38	87.31		20.13
10	Koppu et al. (22)	Fitness Oriented Dragonfly Optimization algorithm (F-DA)	Cleveland	84.44	30.98	51.16	87.96	38.59	15.56
			Statlog	86.5	38.42	**91.2**	86.05	54.07	13.5
11	Tama et al. (23)	Two tier ensemble method using RF, gradient boosting and XGBoost Classifiers and PSO	Cleveland	85.71				86.49	14.29
			Hungarian	91.18				**90.91**	8.82
12	Amin et al. (24)	Vote with NB and LR		87.41					12.59
13	Mohan et al. (21)	HRFLM	Cleveland	88.4	90.1	**92.8**	82.6	90	11.6

PSO, particle swarm optimization; CE, classification error; CART, classification and regression tree; MTE, multi-tier ensemble; RF, random forest; GA, genetic algorithm; RFE, recursive feature elimination; NB, Naïve Bayes; LR, logistic regression; and HRFLM, hybrid random forest with a linear model. The bold values suggest the performance is marginally better than the proposed method.

## 6 Conclusion and future scope

In spite of remarkable advancements in medical technology, HD continues to be a leading cause of death. This is partly due to the lack of professional support when using new diagnostic instruments. To address this issue, the authors proposed a novel and robust diagnostic framework for the accurate and early prediction of HD, based on the combination of improved PSO with LR, LDA, SVM, GNB, and XGBoost Classifier. The IPSO, enhanced with dynamic inertia weight and a mutation operator, effectively overcomes the premature convergence of standard PSO, leading to more optimal feature subsets and hyperparameter settings. The proposed model was employed on a combination of five datasets, i.e., Cleveland, Statlog, Hungarian, Switzerland, and Long Beach datasets, and the results were promising. The experimental outcomes highlight that the proposed model not only achieves commendable performance metrics on the training dataset but also demonstrates exceptional generalization performance on the validation dataset with fewer classification errors. The ablation study and statistical significance tests (*p*-value <0.05) provided concrete evidence of the individual and combined contributions of our advanced preprocessing suite and the IPSO optimizer. Additionally, the proposed approach achieves competitive or even better performance compared to currently developed methods documented in the literature. These outcomes suggest that the proposed model could be a valuable tool for clinicians in making better and more truthful decisions during the diagnosis of HD. Despite the strong results, the study has some limitations. The framework was tested on tabular clinical data; its performance on unstructured data like echocardiogram images or raw ECG signals was not explored. Furthermore, the interpretability of the complex IPSO-XGBoost model, while highly accurate, could be a hurdle for clinical adoption. In the future, the authors aim to:

- Implement and compare various other nature-inspired optimization techniques (e.g., Gray Wolf Optimizer, Whale Optimization Algorithm) for feature selection to evaluate how well they perform when combined with traditional ML classification methods.
- Develop explainable AI (XAI) techniques, such as SHAP (SHapley Additive exPlanations), to provide clear explanations for the model's predictions, thereby increasing trust and usability among medical professionals.
- Apply the proposed hybrid model to investigate multiclass classification problems (e.g., classifying different types of heart disease) and validate its performance on a wider range of medical datasets.
- Explore the integration of deep learning models and the use of data from wearable IoT devices for real-time, continuous heart disease monitoring.

## Data Availability

Publicly available datasets were analyzed in this study. This data can be found at: https://doi.org/10.24432/C52P4X.
